# Repetitive Transcranial Magnetic Stimulation over Right PMd Does Not Affect the Stability of Rhythmic Bimanual Finger Movements

**DOI:** 10.1523/ENEURO.0026-26.2026

**Published:** 2026-08-21

**Authors:** Ronan Denyer, Anjana Rajendran, Cristina Rubino, Lara A. Boyd

**Affiliations:** ^1^Institute of Neuroscience, Université Catholique de Louvain, Woluwe-Saint-Lambert, Brussels 1200, Belgium; ^2^Graduate Program in Neuroscience, University of British Columbia, Vancouver, British Columbia V6T1Z3, Canada; ^3^Department of Physical Therapy, University of British Columbia, Vancouver, British Columbia V6T1Z3, Canada; ^4^Graduate Program in Rehabilitation Sciences, University of British Columbia, Vancouver, British Columbia V6T1Z3, Canada; ^5^Centre for Vision Research, York University, Toronto, Ontario M3J 1P3, Canada

**Keywords:** action–perception coupling, bimanual coordination, dorsal premotor cortex, human, motor control, transcranial magnetic stimulation

## Abstract

How the brain controls rhythmic asymmetric bimanual movements remains an enduring question in neuroscience. Prior research suggests that the right dorsal premotor cortex (PMd) is essential for maintaining asymmetric bimanual rhythmic finger-tapping patterns. However, such findings have not fully accounted for evidence showing that asymmetric movements impose greater cognitive control demands than symmetric ones. Thus, previously observed disruptions following transient disruption of right PMd may reflect impaired cognitive control rather than motor processes specific to asymmetry. To test this, we manipulated cognitive control demands during bimanual tapping by having participants synchronize movements with regularly flickering visual stimuli. Switching from spatially congruent to symbolic cues reduces tapping stability similarly to switching from symmetric to asymmetric patterns, providing a platform to test the cognitive control hypothesis. Right PMd was targeted with 1 Hz repetitive transcranial magnetic stimulation (rTMS) intended to transiently reduce excitability. Participants (15 females, 13 males) completed symmetric and asymmetric tapping tasks across multiple frequencies. Performance was compared with a sham session. Contrary to expectations, rTMS-related alterations in tapping stability and accuracy were not dependent on movement symmetry or cognitive control demands. Instead, no detectable behavioral effect of rTMS was observed under the present stimulation and testing conditions. These results indicate that asymmetric rhythmic tapping can be maintained after rTMS over right PMd, although lacking direct measures of PMd target engagement limits conclusions regarding cortical perturbation effectiveness. Future work incorporating concurrent neural measures can determine the mechanisms underlying this null effect.

## Significance Statement

How the brain coordinates two-handed movements remains a fundamental question in neuroscience with clinical consequences. The study tests whether the right dorsal premotor cortex (PMd) is responsible for programming asymmetric movements or if it broadly manages the cognitive control demands of such movements. By using noninvasive brain stimulation to challenge right PMd during bimanual movements, we found that bimanual stability remained unaffected regardless of movement symmetry or cognitive control demands condition. These findings indicate that, under the present stimulation and testing conditions, transient challenge to right PMd was not sufficient to disrupt performance. The results highlight the resilience of bimanual coordination while also emphasizing the need for concurrent neural measures when interpreting null effects of repetitive transcranial magnetic stimulation on complex behavior.

## Introduction

Despite decades of research, how the human brain coordinates asymmetric bimanual movements remains an enduring question in motor neuroscience. Research using transcranial magnetic stimulation (TMS) has indicated that the dorsal premotor cortex (PMd) plays a central role in controlling rhythmic asymmetric movements. For example, disrupting the right PMd with single-pulse TMS increases involuntary switching to symmetrical patterns (Meyer-Lindenberg et al., 2002; van den Berg et al., 2010), an effect not seen when targeting the left PMd, bilateral SMA, or S1 (Meyer-Lindenberg et al., 2002). Furthermore, interhemispheric inhibition (IHI) between PMd and M1 is modulated during the preparation of asymmetric movements, and individual differences in bimanual stability correlate with the degree of PMd–M1 IHI modulation (Liuzzi et al., 2011; Fujiyama et al., 2016). Together, these findings suggest PMd may play a specialized role in asymmetric control, possibly via direct connections with corticospinal neurons in M1.

**Figure 1.**
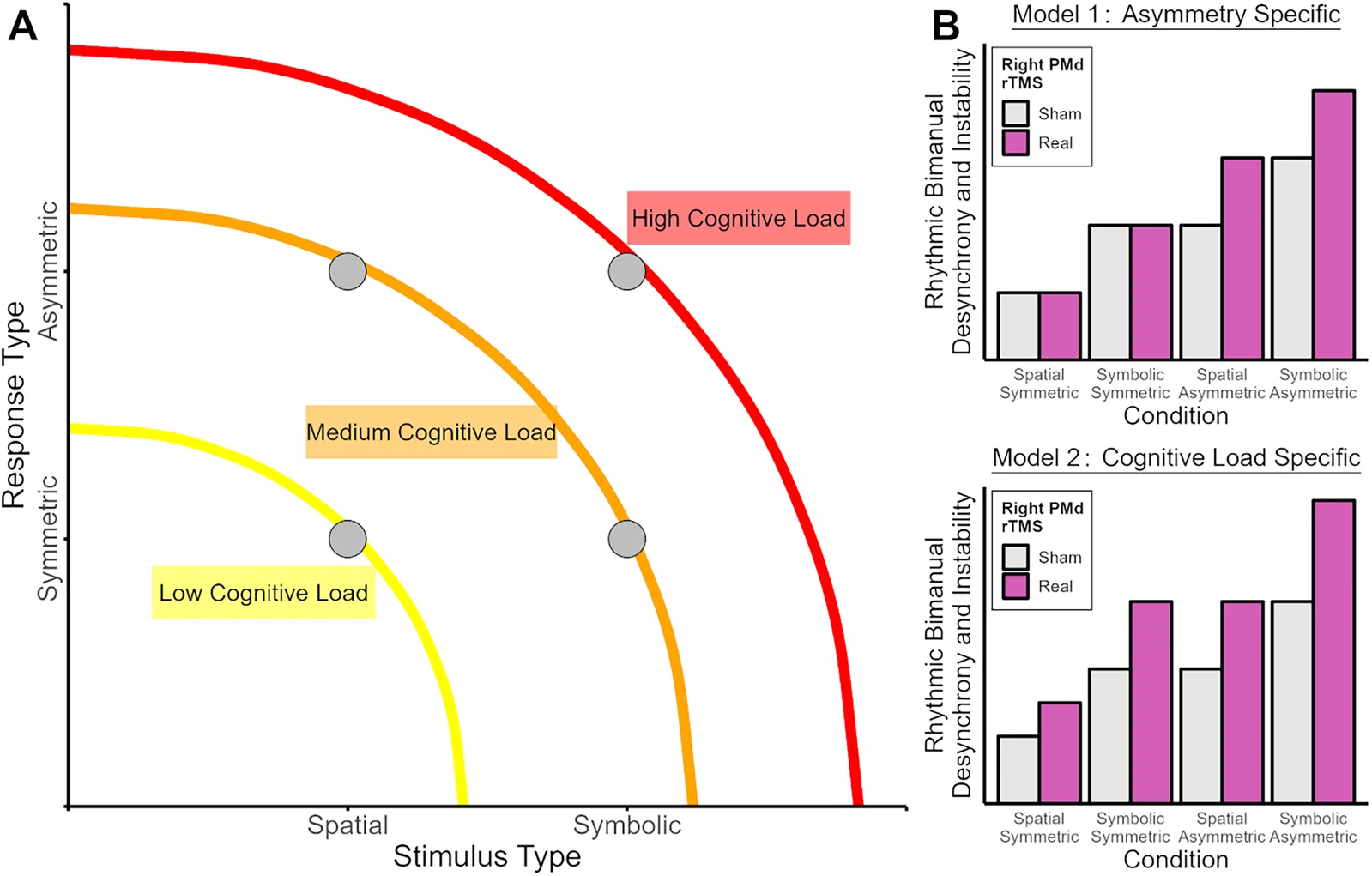
Hypotheses representation. ***A***, Changes in response type from symmetric to asymmetric and changes in stimulus type from spatial to symbolic are associated with increases in cognitive control demands during rhythmic bimanual finger tapping. These factors linearly combine to create four task conditions with varying degrees of cognitive control demands. Expressing task conditions in cognitive control demands space demonstrates the capacity to disentangle response asymmetry from general increases in cognitive control demands across response types. ***B***, Hypothesized outcomes of application of 1 Hz rTMS to rhythmic bimanual finger tapping stability if right PMd is responsible for programming asymmetric response types (top) or if right PMd is responsible for managing cognitive control demands (bottom). For visual simplicity, the schematic illustrates the hypothesized contributions of response type and cue type to cognitive/control demands. Movement frequency is not depicted but was included as an additional task-demand manipulation expected to increase control demands and amplify asymmetry-related costs at higher frequencies.

While PMd is clearly important for asymmetric control, its specific function remains elusive. Beyond the kinematic differences, asymmetric bimanual task demands incur higher cognitive control demands than symmetric ones. For example, asymmetric bimanual movements are destabilized more than symmetric movements by secondary tasks (Summers et al., 1998; Temprado et al., 1999). Similarly, the stability of asymmetric bimanual circling movements is significantly increased when movement frequency is communicated with spatially congruent visual stimuli compared with spatially incongruent stimuli (Byblow et al., 1999). Spatial congruency is associated with decreased perceptual and cognitive control demands in reaction time (RT) paradigms (Diedrichsen et al., 2001, 2003, 2006; Hazeltine et al., 2003; Stanciu et al., 2017; Hesse et al., 2020; Janczyk et al., 2022), as well as in rhythmic finger tapping (Denyer and Boyd, 2025) and finger circling (Wenderoth et al., 2009) paradigms. This raises the possibility that PMd contributes to asymmetric control not solely because it is specialized for programming asymmetry but because it more broadly contributes to the management of cognitive/control demands across task contexts (Fig. 1; Thura et al., 2022; Chouinard and Paus, 2006).

More broadly, the present task also sits within established frameworks of bimanual coordination emphasizing stability constraints and symmetry biases. Symmetric bimanual movements are generally more stable than asymmetric movements, particularly as movement frequency increases, and asymmetry-related performance costs have often been interpreted in terms of neural cross talk or increased control demands required to suppress interference between the two hands and maintain independent limb actions (Summers et al., 1998; Byblow et al., 1999; Temprado et al., 1999; Mechsner et al., 2001; Wenderoth et al., 2009). From this perspective, asymmetric coordination at higher movement frequencies provides a useful context in which to test whether transient challenge to right PMd disproportionately affects performance when coordination stability is placed under greater strain.

To probe the possibility that right PMd manages increased cognitive control demands during asymmetric bimanual behavior, we used 1 Hz repetitive TMS (rTMS) with the aim of transiently reducing excitability in right PMd (Chen et al., 1997; Hoffman et al., 2000; Gerschlager et al., 2001; Fitzgerald et al., 2002) before the performance of a bimanual rhythmic finger tapping task. Right PMd was chosen as the rTMS target as detriments to asymmetric bimanual rhythmic tapping have been shown to be stronger after challenge to right PMd compared with left PMd (Meyer-Lindenberg et al., 2002; van den Berg et al., 2010), and increased brain activation has been shown in the right hemisphere during performance of asymmetric bimanual movements (Wenderoth et al., 2005; Aramaki et al., 2006). Performance was compared against a second sham rTMS session. Different versions of the task were performed, with manipulations to the required movement patterns (symmetric/asymmetric) and the stimulus quality for the cues used to denote the required movement frequency (spatial/symbolic). Spatially congruent stimuli have previously been shown to improve temporal coupling accuracy similarly for symmetric and asymmetric movement patterns (Denyer and Boyd, 2025). Accordingly, in the current context, symbolic cueing is interpreted as increasing cognitive/control demands relative to spatially congruent cueing. Manipulating both movement symmetry and stimulus type therefore provides a platform to test two competing models: (Model 1) PMd is critical for programming asymmetric movements, meaning that challenge to PMd will impair performance exclusively in asymmetric tasks, regardless of the stimulus type (Fig. 1*B*, top), and (Model 2) PMd manages cognitive/control demands during bimanual rhythmic tapping, meaning that disruption of PMd will be associated with detriments that scale with total cognitive control demands (Symbolic-Asymmetric > Symbolic-Symmetric/Spatial-Asymmetric > Spatial-Symmetric; Fig. 1*B*, bottom). Manipulating movement frequency provides an additional task-demand manipulation. Higher movement frequencies increase the control demands of rhythmic tapping and amplify the behavioral costs associated with symbolic cueing and asymmetric coordination, thereby providing a more stringent test of whether challenge to PMd will impair performance under high-demand conditions. Our primary hypotheses were as follows: (H1) tapping accuracy and stability will decrease after real rTMS, following the scaling pattern described by the cognitive control demands specific model; (H2) these effects will be specific to rhythmic behaviors, with no change in a bimanual choice RT task (O'Shea et al., 2007a). To supplement the primary hypotheses, we further predicted that there will be no change in simple RT (H3) or M1 corticospinal excitability (H4), ensuring any observed effects are not attributable to impaired motor execution or direct modulation of M1.

## Materials and Methods

### Participants

A sample size of 28 participants was targeted based on a power analysis of a previous study that deployed a similar behavioral paradigm. This power analysis indicated a sample of 20 participants was well powered (1−*β* = 0.99) to detect a large effect (Cohen's *d* > 0.8) in the two primary interactions of interest (movement frequency/response type interaction and movement frequency/stimulus type interaction). Eight additional participants were targeted due to the addition of an additional fixed effect in the current study (sham stimulation/1 Hz rTMS). Thus, the present study was designed primarily to detect relatively large effects and was less well suited to rule out smaller or more moderate stimulation-related influences. A total of 33 individuals were recruited from Vancouver, British Columbia, to participate in the current study. All participants were right-handed according to the Edinburgh Handedness Inventory and had no history of neurological disease or injury. The majority of participants were recruited via a University of British Columbia undergraduate human research participants pool and received academic credit in exchange for participation. Other participants received financial remuneration for participation. Participation involved a short online introductory session as well as two in-person experimental sessions. Of the 33 recruited participants, 5 dropped out of the study after the first in-person session. These participants were excluded from final analyses, leaving a total of 28 participants (22.9 ± 7 years old; 15 females, 13 males). All protocols were approved by the Clinical Research Ethics Board at the University of British Columbia. Prior to participating, individuals provided written informed consent in accordance with the Declaration of Helsinki.

### Experimental design

**Figure 2.**
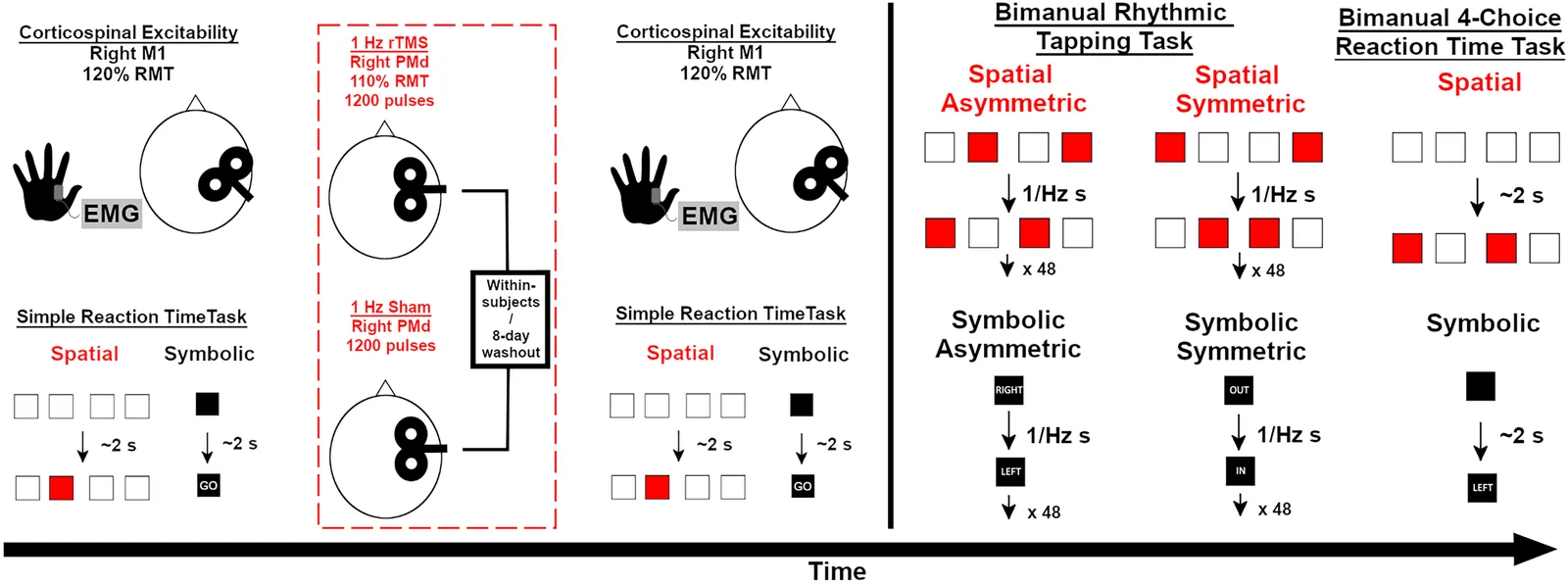
Experimental overview. Corticospinal excitability and simple RT were assessed directly before and after 1 Hz rTMS. All participants received real and sham rTMS across two sessions separated by at least 8 d, with session order counterbalanced between participants. After TMS, participants completed four versions of a bimanual four-finger rhythmic tapping task, followed by two versions of a bimanual four-choice RT task. Within the rhythmic tapping task, the cue type order was counterbalanced between participants, whereas the response type order was fixed such that asymmetric conditions were completed before symmetric conditions. Within each condition, movement frequency always increased from 1.4 to 2.2 to 3 Hz. Within the bimanual four-choice RT task, the stimulus type condition order was counterbalanced between subjects.

**Figure 3.**
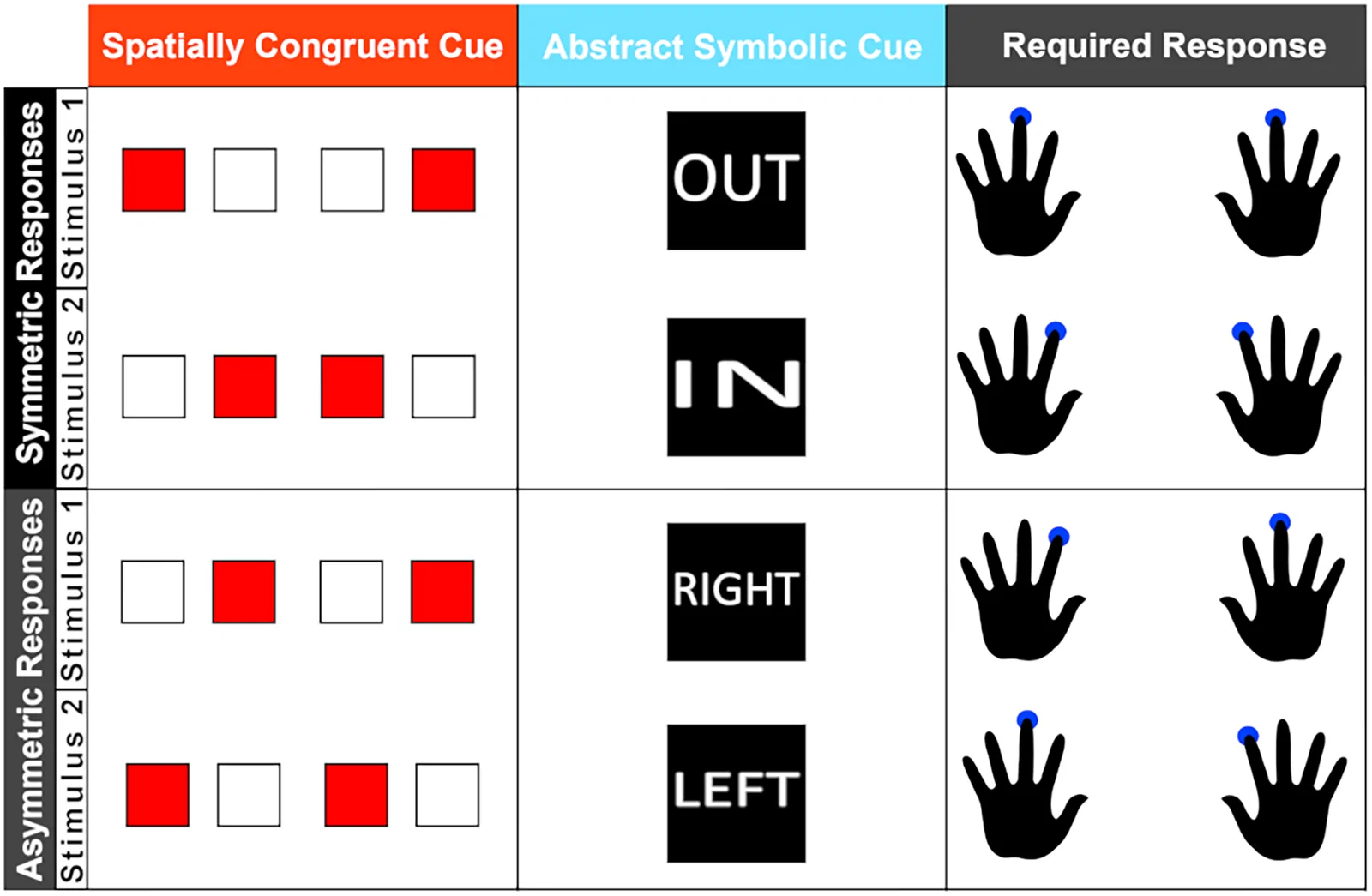
Stimulus–response mappings. In both tasks, movement timing was either cued with spatially congruent visual stimuli, where red boxes directly denoted the relative spatial location of the fingers required to move, or abstract symbolic stimuli where words abstractly denoted the identity of the fingers required to move. Required responses were either symmetric or asymmetric. In the bimanual four-finger rhythmic tapping task, participants performed four versions of the task, in which movement frequency was cued with either spatially congruent or abstract symbolic visual cues, and the required response type was either symmetric or asymmetric. Simple RT task stimulus–response mappings shown in Extended Data Figure 3-1.

Using a within-subject design, all participants completed two experimental sessions to examine the effect of 1 Hz rTMS over right PMd on the control of discrete and rhythmic bimanual movements (Fig. 2). All participants underwent sham and real rTMS across two sessions separated by at least 8 d. The order of sham/real rTMS sessions was counterbalanced between participants. Corticospinal excitability and simple RT were assessed before and after sham/real rTMS. After sham/real rTMS, participants performed four versions of a four-finger bimanual rhythmic tapping task. Task versions differed in terms of whether symmetric or asymmetric tapping patterns were required and whether movement frequency was cued with abstract symbolic stimuli or spatially congruent stimuli (Fig. 3). Next, participants performed two versions of a four-finger bimanual choice RT task: one in which responses were cued with abstract symbolic stimuli, and another in which responses were cued with spatial stimuli. For both task types, the order of task stimulus type (symbolic/spatial) was counterbalanced between participants.

10.1523/ENEURO.0026-26.2026.f3-1 Figure 3-1 Simple reaction time task stimulus-response mappings. Participants performed unimanual simple reaction time trials with the left and right hands, as well as bimanual trials. Trials were collected in separate blocks using either spatially congruent or abstract symbolic cues. Download Figure 3-1, TIF file.

### Procedure

Before completing the in-person rTMS sessions, participants completed a 20 min introductory practice session to ensure they understood the task requirements. The practice session was delivered via online platforms, with participants using a web browser on their personal desktop or laptop computer to complete the session. Participants accessed the practice session via a link to Qualtrics, a secure online survey tool (qualtrics.com), where they first indicated they met the study criteria, digitally signed a consent form, and provided demographic information. Participants were then redirected to the Gorilla Experiment Builder (Anwyl-Irvine et al., 2020; www.gorilla.sc). Participants could not progress to Gorilla unless they were using a desktop or laptop computer with a keyboard. In Gorilla, participants were given instructions on how to correctly perform trials of the four-finger bimanual choice RT task and the bimanual rhythmic finger tapping task. In order to advance, participants had to demonstrate they understood the four stimulus–response mappings for the spatially cued and symbolically cued task versions. They also had to complete six practice trials of each task without making an error.

For the in-person rTMS sessions, participants first completed a TMS safety questionnaire to ensure they were safe to participate in the experiment. Before the first rTMS session, the experimenter verified that the participant had completed the online practice session and that they understood the task requirements. Participants had the opportunity to practice the tasks again under supervision of the experimenter.

Once they had indicated that they understood the task requirements, participants completed a simple RT task. Participants performed symbolically cued and spatially cued versions of the simple RT task on a desktop personal computer with a wired keyboard and a widescreen high-definition monitor (BenQ; 144 Hz refresh rate). Participants were shown a fixation stimulus followed by an imperative stimulus indicating they should respond as fast as possible (see Extended Data Fig. 3-1 for detailed stimulus–response mappings). Participants were explicitly told which finger(s) to respond with before each block of trials. A 2–4 s intertrial interval was randomly inserted between trials to prevent participants from anticipating the onset of the imperative stimulus. For each task version, participants completed a block of six unimanual trials with their left index finger, a block of six unimanual trials with their right index finger, and a block of six bimanual trials with their left and right index fingers (36 trials total).

Next, single-pulse TMS was applied over M1 to establish resting motor threshold and corticospinal excitability. Surface electromyography (EMG) was recorded using bipolar electrodes placed over bilateral FDI muscles. A ground electrode was placed over the ulnar styloid process of the right arm. EMG was sampled at 5,000 Hz, amplified 1,000 times, and bandpass filtered (50–450 Hz; Delsys). EMG recording was controlled using the VETA MATLAB toolbox (Jackson and Greenhouse, 2019) and displayed on a monitor outside of the participant’s field of vision. TMS was administered using a Magstim Rapid2 stimulator with an a AirFilm Coil. Brainsight neuronavigation (Rogue Research) was used to ensure coil positioning was accurate and consistent across sessions. An infrared tracker was attached to the TMS coil which allowed experimenters to control coil position relative the head in real-time throughout each session.

For TMS over M1, the center of the coil was positioned in a posterior-to-anterior orientation 45° to the midsagittal plane over the right M1. The FDI hotspot was defined as the scalp position at which single-pulse TMS most consistently elicited the largest MEPs in the resting FDI. Once the M1 hotspot was identified, resting motor threshold was determined as the minimum stimulation intensity required to elicit MEPs with a peak-to-peak amplitude of at least 0.05 mV in 5 out of 10 consecutive trials (see Table 1 for individual resting motor threshold values). After establishing resting motor threshold, corticospinal excitability was assessed by eliciting 10 MEPs over the M1 hotspot at an intensity of 120% of resting motor threshold.

**Table 1.** Participant characteristics (participant sex, age, and resting motor threshold values)

Participant	Sex	Age	RMT—SHAM	RMT—REAL
1	M	27	45	46
2	F	20	51	52
3	F	34	55	53
4	F	39	71	63
5	F	30	48	51
6	F	19	39	37
7	F	22	42	40
8	M	19	75	77
9	M	19	70	70
10	M	19	54	54
11	M	20	60	62
12	F	20	35	39
13	M	19	64	61
14	F	20	59	58
15	M	19	60	60
16	M	23	54	57
17	F	18	36	36
18	F	18	60	59
19	F	21	55	54
20	F	22	36	39
21	F	18	51	56
22	M	19	59	57
23	M	20	36	34
24	F	19	57	55
25	M	18	71	70
26	F	22	51	47
27	M	33	67	62
28	M	45	48	48

For rTMS over right PMd, the center of the coil was positioned in a lateral-to-medial orientation 90° to the midsagittal plane. The coordinates for PMd stimulation were established by moving the coil 7% of the distance from the nasion to the inion anterior from the “hotspot” and 3.5% of the distance from the nasion to the inion medial to the “hotspot” (O'Shea et al., 2007a, b). Stimulation intensity during rTMS over PMd was set to 110% of resting motor threshold (Meehan et al., 2013). Before beginning rTMS, five single pulses of TMS were applied over the PMd target at 110% of resting motor threshold to test whether any MEPs in M1 were elicited. If MEPs of any amplitude were observed, TMS intensity was iteratively lowered by 1% maximum stimulator output until five consecutive trials without MEPs were observed.

For sham rTMS sessions, the AirFilm Coil was switched to a sham coil which looked and sounded like the AirFilm Coil. rTMS took a total of 20 min, with 1,200 pulses delivered at a rate of 1 Hz. Participants were instructed to avoid falling asleep, avoid moving their hands, and inform the experimenter if they experienced any discomfort.

After rTMS was completed, corticospinal excitability assessments were repeated by delivering 10 single pulses of TMS over M1 at 120% of resting motor threshold. Next, participants repeated the simple RT task as described above. After these assessments, participants started the four-finger bimanual rhythmic tapping task. For each task version, participants performed three blocks of 48 taps. Before the start of each block, a fixation cross was displayed with a clock graphic counting down from 3 s to allow participants to anticipate the start of the block. There was an opportunity for a break after every block. The movement frequency of rhythmic tapping started at 1.4 Hz and increased by 0.8 Hz each block, finishing at 3 Hz. The order of cue type (symbolic/spatial) was counterbalanced between participants. However, response type was not counterbalanced: all participants completed the asymmetric conditions before the symmetric conditions. Within each condition, movement frequency always increased from 1.4 to 2.2 to 3 Hz. This ordering was chosen to avoid beginning participants at the highest movement frequency and to ensure that the asymmetric conditions, which were of greatest theoretical interest, were completed earlier in the poststimulation period.

Upon completion of the four-finger bimanual rhythmic tapping task, participants began the four-choice bimanual RT task. Participants performed two versions of the task (symbolic stimuli/spatial stimuli) in alternating blocks, with the order of task version counterbalanced. There were four stimulus–response mappings for the symbolic and spatial versions of the task (Fig. 3). For each task version, participants performed four blocks of 12 task trials each (12 per each four stimulus–response mappings) with an opportunity for a break every block. The task version was alternated every two blocks, and the order of the four-choice RT stimuli was randomized between participants and between task versions. A random intertrial interval of 2–4 s was used so that participants could not anticipate stimulus onset timing. RT and the identity of keys pressed were recorded on each trial.

To clarify the precise timing of poststimulation behavioral testing, we quantified the interval between the end of stimulation and the onset of each poststimulation task. The first poststimulation assessment began within ∼2 min of stimulation offset, reflecting the time required to collect 10 poststimulation MEPs at 5 s intervals and to adjust the setup before the simple RT task. Mean task-onset latencies were similar between sessions: simple RT began at 2.0 min, rhythmic bimanual tapping began at 5.0 min, and bimanual choice RT began at 12.9 min after rTMS, compared with 2.0, 5.0, and 13.1 min after sham stimulation, respectively. Timing was similar between sessions and differed little between participants (Table 2).

**Table 2.** Mean latency to poststimulation task onset and total poststimulation testing duration

Stimulation type	Simple RT latency, min	Bimanual tapping latency, min	Choice RT latency, min	Total testing duration, min
rTMS	∼2	5.0 (4.7–5.8)	12.9 (11.9–15.1)	24.1 (22.2–27.5)
Sham	∼2	5.0 (4.7–6.9)	13.1 (12.0–17.0)	24.3 (22.6–27.5)

Values are reported in minutes as mean (range). Latencies incorporate an estimated 2 min interval for the poststimulation MEP assessment.

After their second rTMS session, participants completed an exit questionnaire in which they were asked if they used any explicit strategies during the four-finger bimanual rhythmic tapping task and whether they noticed any difference in the rTMS between sessions. Next, the experimenter informed the participant that one of the sessions had used real rTMS and the other had used sham rTMS. To assess explicit knowledge, participants were asked if they noticed any differences in the stimulation between the sessions and then to guess which day was real rTMS. No participant gave a response indicating awareness that one of the sessions was sham rTMS. Furthermore, at the group level, participants did not perform above chance at identifying which session was real rTMS, indicating there was no systematic explicit awareness of when real or sham rTMS was applied.

### Data processing

Custom MATLAB (version: 9.6.0.1150989, R2019a) scripts were used to process data downloaded from Gorilla. For the simple and choice RT tasks, RT for each individual trial was extracted from Gorilla. For bimanual trials, the mean of RT values for each responding finger was calculated to create a single bimanual RT value for each bimanual trial. For each condition of the choice RT task, RT values greater than three scaled median absolute deviations were defined as outliers and removed from further analysis to prevent positive RT distribution skew from affecting mean and standard deviation (SD) RT calculations (Lachaud and Renaud, 2011). Choice RT trials were designated as an error if the wrong pair of keys was pressed, if no keys were pressed, if only one key was pressed, or if more than two keys were pressed.

For the four-finger bimanual rhythmic tapping task, an array of key press timing values was filtered from the raw data file for each finger and for each block. These arrays were used to calculate a relative phase value for each key press made. Relative phase values were calculated according to the following formula:

$$\phi =\frac{t_{\mathrm{Tar},i}-t_{\mathrm{Ref},i}}{t_{\mathrm{Ref},i+1}-t_{\mathrm{Ref},i}}\times 360^{\circ }.$$

**Figure 4.**
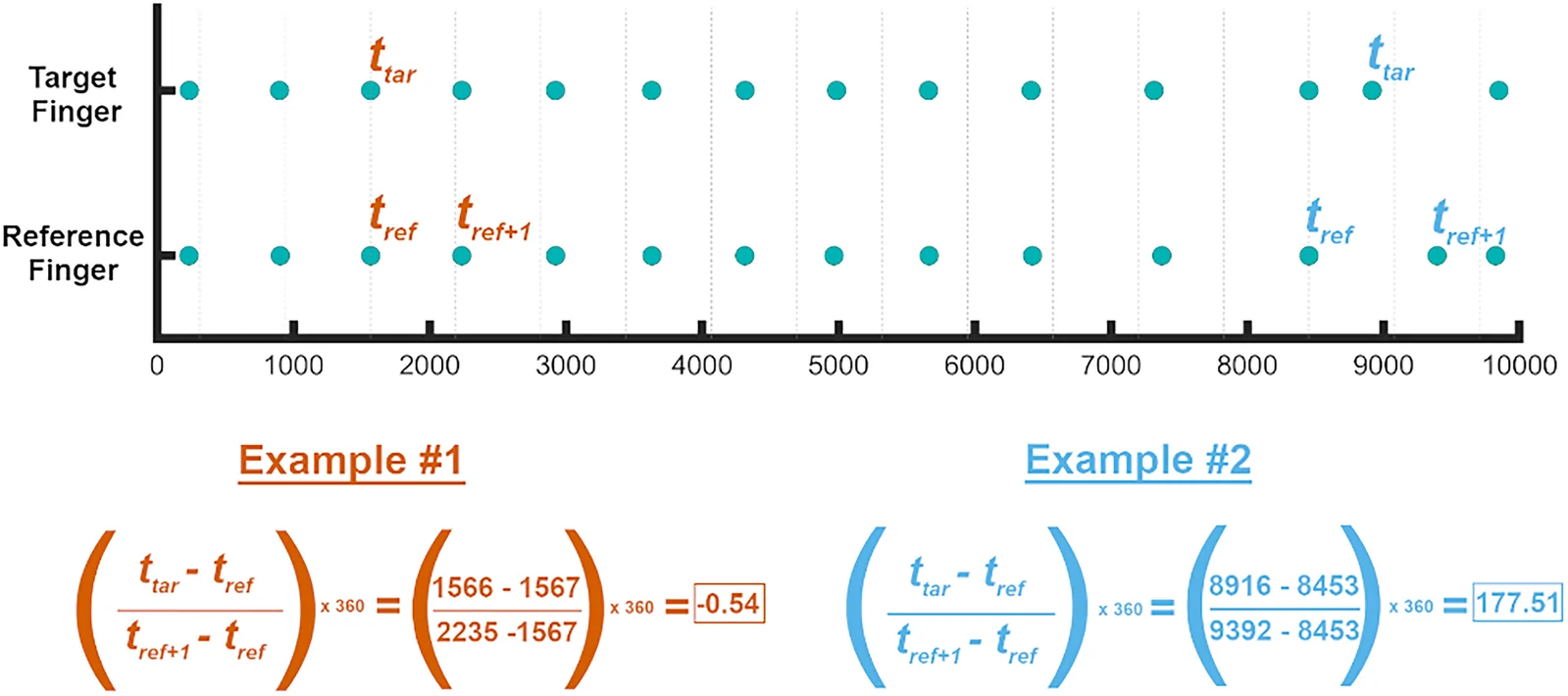
Method to calculate relative phase of bimanual coupling from key press data. An array of bimanual key press times from one task block is shown for target and reference fingers. The difference in time between each target finger key press and the temporally nearest reference key press is compared with the difference in time between that reference finger key press and the following reference finger key press. When bimanual temporal coupling is high, relative phase values are low, as is shown in Example #1. When bimanual temporal coupling is low, relative phase values are high, as is shown in Example #2.

Here *ϕ* equals relative phase, *t*_tar,i_ equals the time of the *i*th tap of the target digit, *t*_ref,i_ equals the time of the *i*th reference tap, and *t*_ref,i_ _+_ _1_ equals the time of the *i*th +1 reference tap (Carson, 1995; Kodama et al., 2015; see Fig. 4 for a graphical example). For symmetric blocks, the reference finger was the same effector on the opposite hand, while for asymmetric trials, the reference finger was the opposite finger of the opposite hand. The first four key presses were excluded from relative phase calculations, to allow participants time to get in rhythm.

To facilitate a supplementary analysis of the effects of rTMS on within-hand coordination stability, within-hand intertap interval (ITI) variability was quantified from the key press timing values for the 3 Hz rhythmic tapping blocks. For each finger and each block, the SD of the ITI was calculated and normalized to the ideal ITI for that block. Normalized ITI variability values were then averaged across fingers within each hand, yielding separate within-hand variability measures for the left and right hands.

Offline analysis of TMS/EMG data was performed using the VETA toolbox (Jackson and Greenhouse, 2019) and custom-automated procedures within MATLAB. MEP peak-to-peak amplitude was the sole EMG variable of interest. The “findEMG.m” function was used to automatically identify EMG events, which were visually inspected by a human rater using the “visualizeEMG.m” function. Adjustments were made to MEP onset and offset when necessary, using the “visualizeEMG.m” function. Any trials that contained >0.05 mV of background muscle activity in either hand 50 ms prior to the TMS artifact were removed from further analysis (<0.5% of MEPs collected were removed).

### Dependent variables

#### Four-finger bimanual rhythmic tapping task

1. *Tapping synchrony*—The mean absolute timing error was calculated from relative phase to provide a measure of tapping synchrony. Absolute timing error was calculated for each key press by subtracting the relative phase of each key press from ideal relative phase (0°) and calculating the absolute value of the resultant integer. The mean of absolute timing error was then calculated for each block.
2. *Tapping stability*—The SD of absolute error was calculated for each block to provide a measure of tapping stability.
3. *Transition occurrence*—Blocks in which a transition key press occurred (i.e., a key press with absolute relative phase greater than 180°) were marked as having a transition occurrence (1), whereas blocks in which no transition key press occurred were marked as having no transition occurrence (0).
4. *ITI variability*—The SD of ITIs was calculated for each finger, normalized to the ideal ITI for that block, and averaged across fingers within each hand to provide a measure of within-hand ITI variability.

#### Four-finger bimanual choice RT task

1. *RT*—mean RT for symmetric and asymmetric trials was calculated for each stimulus type condition to provide a measure of RT. Error trials were not included in mean calculations.
2. *RT variability*—the SD of RT for symmetric and asymmetric trials was calculated for each stimulus type condition. Error trials were not included in SD calculations.

#### Simple RT task

1. *RT*—mean RT for symmetric and asymmetric trials was calculated for each stimulus type and response condition to provide a measure of RT.

#### Single-pulse transcranial magnetic stimulation

1. Corticospinal excitability—the mean peak-to-peak amplitude of single-pulse MEPs was calculated to provide a measure of corticospinal excitability.

### Statistical analysis

Statistical analyses were carried out in RStudio (version: 2023.06.1). Post hoc analyses were performed using Tukey's correction for multiple comparisons where appropriate.

#### Quality control

All data were visually checked to ensure that statistical models were based on high-quality data. Exclusions were applied at the participant, block, and observation level depending on the nature of the issue. For the four-finger bimanual rhythmic tapping task, one participant did not complete the online practice session before their first in-person experimental session. Therefore, their data from this task were excluded, as the lack of practice may have introduced a confound due to reduced task familiarity in their first session compared with their second session. A further two participants were excluded because they were either pressing the wrong keys or holding down keys on the majority of blocks, meaning that outcome measures could not be calculated reliably. Of the remaining 25 participants, 3 were found to be pressing the wrong keys during tapping on only 1 or 2 of the total 24 blocks. Outcome measures could not be calculated for these blocks; however, because this error in task performance was limited, these participants were retained in the final analysis with partial datasets. Therefore, the final sample size for the four-finger bimanual rhythmic tapping task was 25 participants.

For corticospinal excitability, technical issues with EMG data collection meant that MEP amplitude could not be calculated for two participants. Therefore, the final sample size for the corticospinal excitability analysis was 26 participants.

Shapiro–Wilk tests were used to assess skewness across all conditions for each behavioral dependent measure. For the rhythmic tapping task, these tests showed some degree of skewness in at least one condition for all dependent variables. To reduce the risk of skewness biasing statistical models and in line with previous statistical approaches (Denyer and Boyd, 2025), observations deemed extreme outliers (i.e., 3 SD above or below the mean) were removed from the dataset for each dependent measure. This led to partial datasets for some conditions. For the rhythmic tapping task, removal of extreme outliers resulted in the exclusion of 29 of 595 total observations (∼5% of the dataset) for the mean absolute error of relative phase analysis and 29 of 595 total observations (∼5% of the dataset) for the SD of absolute error analysis. For the supplementary within-hand ITI variability analysis, removal of extreme outliers resulted in the exclusion of 19 of 400 total observations (∼4.8% of the dataset). Outlier detection was performed within each combination of movement frequency, response condition, cue condition, and stimulation condition, and inspection of the removed observations did not indicate concentration in any single task condition. Rerunning analyses without outlier removal produced a similar overall pattern of results. The simple and choice RT tasks showed limited skewness, and therefore no observations were removed from these datasets before inferential analysis.

The presence of some skewness and partial datasets for some task outcome measures further informed the decision to use linear mixed-effects regression (LMER) rather than traditional *F* tests, as LMER is robust to violations of distributional assumptions required by parametric tests (Schielzeth et al., 2020). LMER also accounts for random variation due to participants by modeling multiple intercepts for each participant as a random effect rather than a single mean intercept (Baayen et al., 2008; Nimon, 2012; Magezi, 2015; Boisgontier and Cheval, 2016). The use of LMERs also allows for the inclusion of partial data, meaning that removal of isolated unusable blocks or extreme outlier observations did not necessitate exclusion of the entire dataset for the associated participant. A summary of statistical tests is shown in Table 3.

**Table 3.** Statistical table

Row	Data structure	Type of test	Confidence intervals [95% CI]
**a**	Normal distribution (residuals)	LMER: mean absolute error (rhythmic tapping)	[−8.38, 16.48]
**b**	Normal distribution (residuals)	LMER: SD of absolute error (rhythmic tapping)	[−40.64, 182.72]
**c**	Binomial distribution	Logistic regression: transition probability	[0.02, 72.52]
**d**	Normal distribution (residuals)	LMER: mean RT (choice RT task)	[−39.66, 30.78]
**e**	Normal distribution (residuals)	LMER: mean absolute error (rhythmic tapping, focused 3 Hz supplementary analysis)	[−2.93, 9.15]
**f**	Normal distribution (residuals)	LMER: SD of normalized ITI (rhythmic tapping)	[−0.18, 0.17]
**g**	Normal distribution (residuals)	LMER: SD of RT (choice RT task)	[−31.75, 19.03]
**h**	Normal distribution (residuals)	LMER: mean RT (simple RT task)	[−23.78, 24.77]
**i**	Normal distribution (residuals)	LMER: corticospinal excitability (MEP amplitude)	[−0.37, 0.54]
**j**	Normal distribution	Bayesian repeated-measures ANOVA (mean absolute error)	[−0.77, 0.77] (credible interval)

Data structure denotes the structure of the data; type of test denotes the statistical test deployed; confidence intervals denotes the confidence/credible intervals of the key interaction of interest.

### Primary analyses

#### Four-finger bimanual rhythmic tapping task

LMERs were performed to establish the effect of fixed effects of STIMULATION CONDITION (rTMS/sham), STIMULUS TYPE (symbolic/spatial), RESPONSE TYPE (symmetric/asymmetric), and MOVEMENT FREQUENCY (1.4, 2.2, 3 Hz) on the mean absolute error and the SD of absolute error of relative phase (Hypothesis 1). Participant ID was included as a random intercept.

Given that transition occurrence was a dichotomous variable (0 or 1), logistic regression (LaValley, 2008) was used to model the probability of transition occurrence across categorical factors STIMULATION CONDITION (rTMS/sham), STIMULUS TYPE (symbolic/spatial), RESPONSE TYPE (symmetric/asymmetric), and MOVEMENT FREQUENCY (1.4, 2.2, 3 Hz; Hypothesis 1).

#### Four-finger bimanual choice RT task

LMERs were used to establish the effect of fixed factors STIMULATION CONDITION (rTMS/sham), STIMULUS TYPE (symbolic/spatial), and RESPONSE TYPE (symmetric/asymmetric) on mean RT and the SD of RT (Hypothesis 2). Participant ID was included as a random intercept.

### Supplementary analyses

#### Four-finger bimanual rhythmic tapping task

To determine whether relative phase may mask effects of rTMS on unilateral destabilization in the highest-demand movement frequency condition (3 Hz), an additional LMER was performed on normalized within-hand ITI variability from the 3 Hz blocks only. Fixed effects were HAND (left/right), STIMULUS TYPE (symbolic/spatial), RESPONSE TYPE (symmetric/asymmetric), and STIMULATION CONDITION (rTMS/sham), with Participant ID included as a random intercept.

To provide a more focused test of stimulation effects in the highest-demand movement frequency condition, an additional LMER was performed on mean absolute error data from the 3 Hz blocks only. This follow-up was conducted because the primary omnibus model contained multiple within-subject factors and higher-order interactions, which may have reduced sensitivity to a stimulation effect specific to the highest-demand condition. Fixed effects were RESPONSE TYPE (symmetric/asymmetric), STIMULUS TYPE (symbolic/spatial), and STIMULATION CONDITION (rTMS/sham), with Participant ID included as a random intercept.

#### Simple RT task

LMERs were used to establish the effect of fixed factors STIMULATION CONDITION (rTMS/sham), STIMULUS TYPE (symbolic/spatial), RESPONSE TYPE (left-unimanual/right-unimanual/bimanual), and TIME (pre/post) on mean RT. Participant ID was included as a random intercept.

#### Corticospinal excitability

LMERs were used to establish the effect of fixed factors STIMULATION CONDITION (rTMS/sham) and TIME (pre/post) on corticospinal excitability. Participant ID was included as a random intercept.

### Exploratory analyses

#### Assessing the strength of the null effect using of 1 Hz rTMS on bimanual rhythmic tapping

As we did not find an effect of stimulation condition during bimanual rhythmic tapping, we ran additional statistics to assess whether this discovery was driven by an insufficient sample size; we conducted an exploratory Bayesian repeated-measures ANOVA on the mean absolute error data. Bayesian statistics are useful at assessing the strength of a null effect, as unlike frequentist statistics, the Bayes factor provides an objective metric of how confident one can be in a given null effect (Hoijtink et al., 2019).

The Bayesian repeated-measures ANOVA had identical fixed effects to the a priori LMER described above. It also included Participant ID as a random slope. All Bayesian analyses were performed in JASP (version 0.19).

### Code availability

We intend to openly share study data and analysis code on a public repository upon acceptance of the manuscript.

## Results

### Four-finger bimanual rhythmic tapping task

#### Tapping synchrony

**Figure 5.**
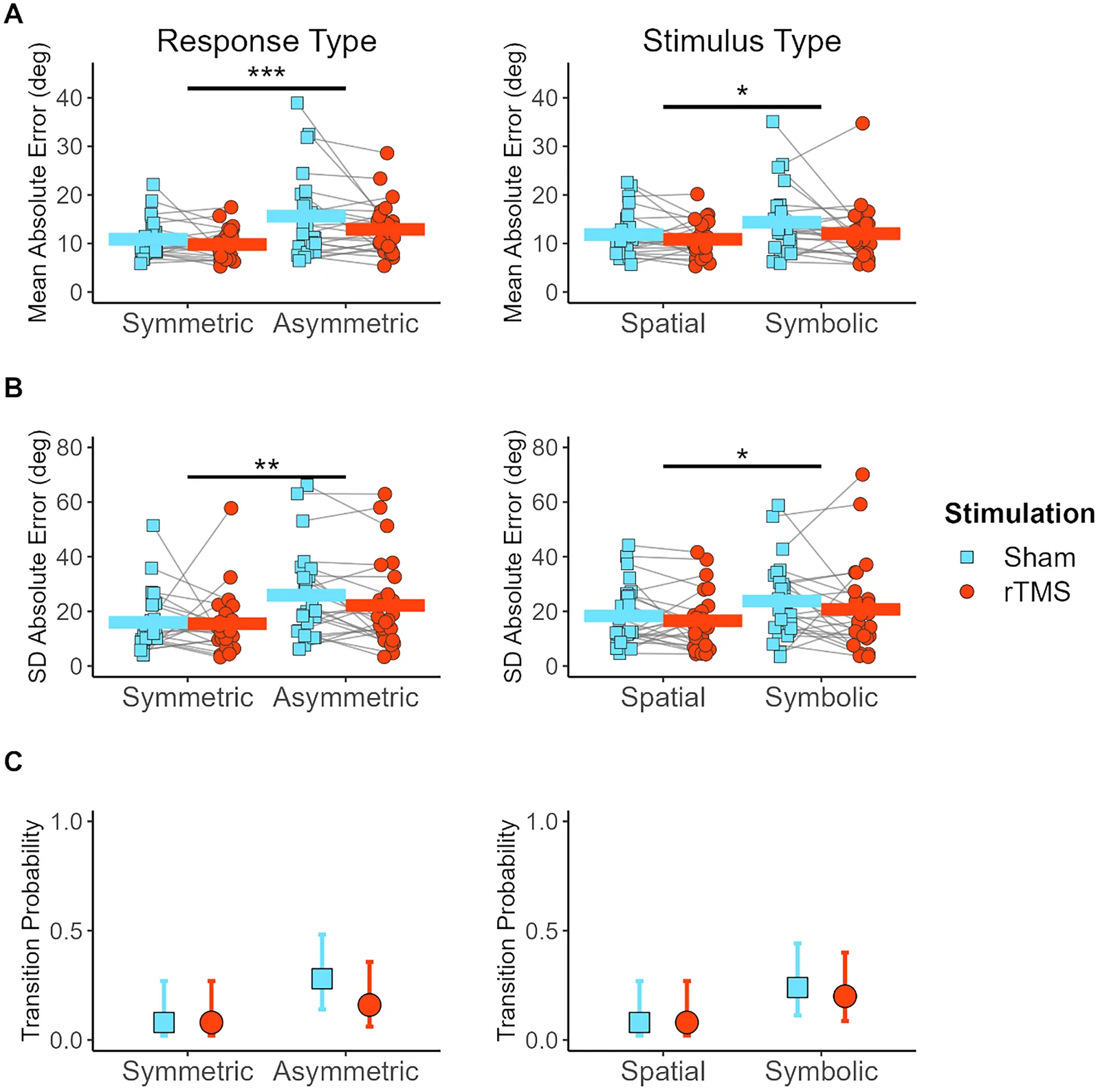
rTMS had no effect on bimanual rhythmic tapping relative to sham. Performance in the rhythmic bimanual tapping task across response type (left column), stimulus type (right column), and stimulation type (colors) conditions. Data are collapsed across levels of movement frequency. ***A***, Mean absolute error of relative phase. Cross bars represent group means, dots represent individual participant means collapsed across movement frequency, and lines link participants across stimulation conditions. ***B***, SD of absolute error. Cross bars represent group means, dots represent individual participants, and lines link participants across stimulation conditions. ***C***, Probability of transition out of required tapping pattern. Dots represent predicted probability based on logistical regression, and error bars represent 95% confidence intervals. Results from additional supplementary analyses shown in Extended Data Figures 5-1 and 5-2. *p < 0.05; **p < 0.005; ***p < 0.0001.

Results from the four-finger bimanual rhythmic tapping task are shown in Figure 5. Mean absolute error increased with movement frequency across task conditions, as was evidenced by a main effect of MOVEMENT FREQUENCY between 1.4 and 3 Hz conditions (*β* = 9.39; 95% confidence interval [CI; 5.02 13.76]; *p* < 0.001). Estimated marginal means indicated that mean absolute error was significantly increased between 1.4 and 2.2 Hz (*µ* = 4.93 ± 0.8; *p* < 0.0001; *d* = 0.64), 1.4 and 3 Hz (*µ* = 13.53 ± 0.79; *p* < 0.0001; *d* = 1.76), and 2.2 and 3 Hz (*µ* = 8.59 ± 0.8; *p* < 0.0001; *d* = 1.12) conditions. The main effect of MOVEMENT FREQUENCY should be considered in the context of a significant MOVEMENT FREQUENCY × RESPONSE TYPE interaction effect (*β* = 9.29; 95% CI [3.08 15.5]; *p* = 0.003), which indicated that the detrimental effects of asymmetric patterns compared with symmetric patterns on mean absolute error significantly increased at 3 compared with 1.4 Hz. Estimated marginal means were calculated to better understand the simple main effects of RESPONSE TYPE across all levels of MOVEMENT FREQUENCY. Asymmetric response types were found to significantly increase mean absolute error compared with symmetric patterns at 3 Hz (*µ* = 8.48 ± 1.12; *p* < 0.0001; *d* = 1.10), but not at 1.4 Hz (*µ* = 0.02 ± 1.12; *p* = 0.99; *d* = 0.00) or 2.2 Hz (*µ* = 2.4 ± 1.13; *p* = 0.28; *d* = 0.31). Surprisingly, there was no significant MOVEMENT FREQUENCY × STIMULUS TYPE interaction effect between 1.4 and 3 Hz conditions (*p* = 0.5). However, estimated marginal means indicated that there was a small but significant general effect of STIMULUS TYPE when collapsing across MOVEMENT FREQUENCY conditions (*µ* = 1.51 ± 0.65; *p* = 0.019; *d* = 0.20). There was also no significant main effect of STIMULATION CONDITION (*p* = 0.99) or any significant interaction effects involving STIMULATION CONDITION (Fig. 5*A*).

10.1523/ENEURO.0026-26.2026.f5-1 Figure 5-1 Focused mean absolute error analysis during 3 Hz movement frequency blocks. Mean absolute error of relative phase at 3 Hz for symmetric and asymmetric response types performed with spatially congruent and symbolic cueing under sham (left) and real rTMS (right) stimulation conditions. Performance at 3 Hz was not measurably impaired by rTMS relative to sham in any task condition. Points represent individual participant mean values, horizontal bars represent group mean values, and grey lines link each participant’s sham and rTMS values within condition. Extreme outliers were removed from each condition. Download Figure 5-1, TIF file.

10.1523/ENEURO.0026-26.2026.f5-2 Figure 5-2 Within-hand intertap interval variability analysis during 3 Hz movement frequency blocks. Normalized intertap interval standard deviation at 3 Hz for the left hand (top) and right hand (bottom) during symmetric and asymmetric response types performed with spatially congruent and symbolic cueing under sham and real rTMS stimulation conditions. Within-hand timing variability was greater in the highest-demand task conditions, but there was no evidence that rTMS selectively destabilized either hand relative to sham. Points represent individual participant mean values, horizontal bars represent group mean values, and grey lines link each participant’s sham and rTMS values within condition. Extreme outliers were removed from each condition. Download Figure 5-2, TIF file.

Because asymmetric tapping costs were greatest at 3 Hz, we conducted a follow-up analysis restricted to the 3 Hz blocks. This focused model revealed no main effect of STIMULATION CONDITION (*β* = −0.04; 95% CI [−2.02, 1.95]; *p* = 0.970), no RESPONSE TYPE × STIMULATION CONDITION interaction (*p* = 0.878), no STIMULUS TYPE × STIMULATION CONDITION interaction (*p* = 0.751), and no RESPONSE TYPE × STIMULUS TYPE × STIMULATION CONDITION interaction (*p* = 0.780). Thus, even in the highest-demand movement frequency condition, there was no evidence that rTMS impaired performance relative to sham (Extended Data Fig. 5-1).

#### Tapping stability

SD of absolute error increased with movement frequency across task conditions, as was evidenced by a main effect of MOVEMENT FREQUENCY between 1.4 and 3 Hz conditions (*β* = 15.48; 95% CI [3.85, 27.11]; *p* = 0.009). Estimated marginal means indicated that the SD of the absolute error was significantly increased between 1.4 and 2.2 Hz (*µ* = 9.81 ± 3.33; *p* = 0.013; *d* = 1.27), 1.4 and 3 Hz (*µ* = 23.53 ± 3.32; *p* < 0.0001; *d* = 3.06), and 2.2 and 3 Hz (*µ* = 13.72 ± 3.3; *p* = 0.0004; *d* = 1.78) conditions. The main effect of MOVEMENT FREQUENCY should be considered in the context of a significant MOVEMENT FREQUENCY × RESPONSE TYPE interaction effect (*β* = 14.6; 95% CI [0.2, 28]; *p* = 0.047), which indicated that the detrimental effects of asymmetric patterns compared with symmetric patterns on the SD of absolute error significantly increased at 3 Hz compared with 1.4 Hz. Estimated marginal means were calculated to better understand the simple main effects of RESPONSE TYPE across all levels of MOVEMENT FREQUENCY. Asymmetric response types were found to significantly increase the SD of absolute error compared with symmetric patterns at 3 Hz (*µ* = 17 ± 2.79; *p* < 0.0001; *d* = 1.10), but not at 1.4 Hz (*µ* = 1.17 ± 2.99; *p* = 0.99; *d* = 0.15) or 2.2 Hz (*µ* = 6.35 ± 2.92; *p* = 0.26; *d* = 0.83). There was no significant MOVEMENT FREQUENCY × STIMULUS TYPE interaction effect between 1.4 and 3 Hz conditions (*p* = 0.86). However, estimated marginal means indicated that there was a significant general effect of STIMULUS TYPE when collapsing across MOVEMENT FREQUENCY conditions (*µ* = 4.5 ± 1.7; *p* = 0.012; *d* = 0.59). There was also no significant main effect of STIMULATION CONDITION (*p* = 0.79) or any significant interaction effects involving STIMULATION CONDITION (Fig. 5*B*).

#### Transition occurrence

Logistic regression analysis revealed that the probability of a transition occurrence did not significantly increase across levels of STIMULATION CONDITION (*p* = 0.99), STIMULUS TYPE (*p* = 0.14), RESPONSE TYPE (*p* = 0.082), or MOVEMENT FREQUENCY (1.4–2.2 Hz, *p* = 0.22; 1.4–3 Hz, *p* = 0.22; 2.2–3 Hz, *p* = 0.99; Fig. 5*C*).

#### ITI variability

The analysis of normalized ITI SD data revealed a significant main effect of RESPONSE TYPE, indicating greater within-hand ITI variability during asymmetric than symmetric tapping (*β* = 0.08; 95% CI [0.02, 0.14]; *p* = 0.009). A significant CUE CONDITION × RESPONSE TYPE interaction was also observed (*β* = 0.11; 95% CI [0.02, 0.19]; *p* = 0.017). Estimated marginal means indicated that symbolic cues significantly increased ITI variability when asymmetric responses were required (*µ* = 0.09 ± 0.02; *p* = 0.0001; *d* = 0.86), but not when symmetric responses were required (*µ* = 0.02 ± 0.02; *p* = 0.34; *d* = 0.25). Notably, there was no main effect of STIMULATION CONDITION (*p* = 0.797), no HAND × STIMULATION CONDITION interaction (*p* = 0.797), and no higher-order interaction involving HAND and STIMULATION CONDITION (all *p* ≥ 0.476). These results indicate that, although within-hand timing variability increased in the highest-demand tapping conditions, rTMS did not selectively destabilize either hand relative to sham stimulation (Extended Data Fig. 5-2).

### Four-finger bimanual choice RT task

#### Mean RT

**Figure 6.**
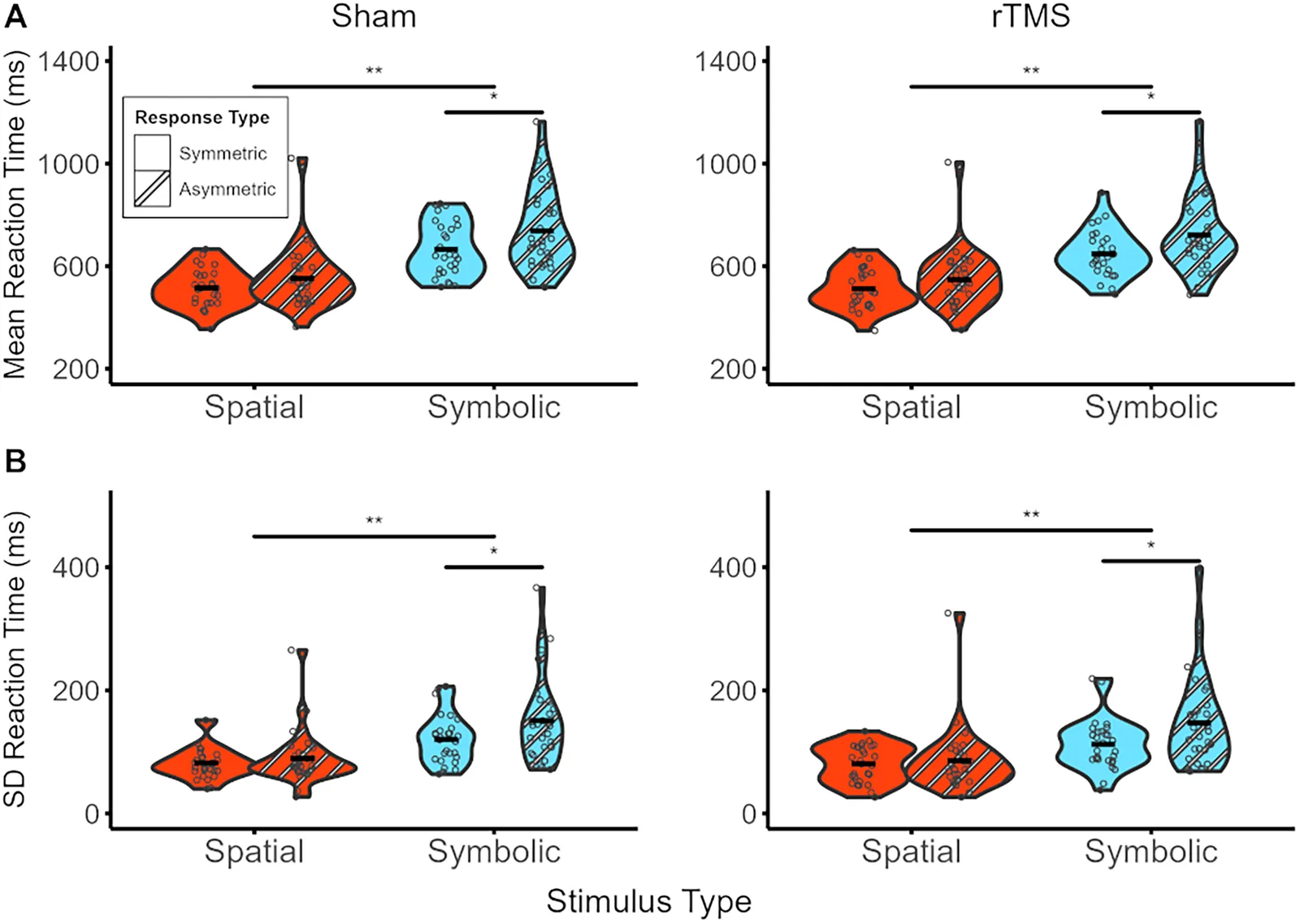
rTMS had no effect on bimanual four-choice RT relative to sham. Mean (***A***) and SD (***B***) of RT values for symmetric and asymmetric response types collected in the spatially cued and symbolically cued bimanual four-choice RT tasks. There was no difference in outcome measures for sham (left) versus real rTMS (right). RT was significantly faster when movements were cued with spatially congruent cues. Furthermore, the cost of asymmetric movements to the magnitude and variability of RT was significant only when movement was cued with abstract symbolic cues. Results from simple RT task shown in Extended Data Figure 6-1. *p < 0.005; **p < 0.0001.

Mean RT was slower when responses were cued with symbolic stimuli compared with spatial stimuli as was evidenced by a significant main effect of STIMULUS TYPE (*β* = 136; 95% CI [110 162]; *p* < 0.001). Similarly, mean RT was slower when asymmetric responses were required compared with symmetric responses, as was evidenced by a significant main effect of RESPONSE TYPE (*β* = 35; 95% CI [3 68]; *p* = 0.033). These main effects should be considered in the context of a significant STIMULUS TYPE × RESPONSE TYPE interaction effect (*β* = 39; 95% CI [14 64]; *p* = 0.003). Estimated marginal means were calculated to further interrogate the interaction effect. Asymmetric responses were found to be significantly slower than symmetric responses when trials were cued with symbolic stimuli (*µ* = 73 ± 15; *p* = 0.0002; *d* = 0.77) but not when cued with spatial stimuli (*µ* = 36 ± 15; *p* = 0.094; *d* = 0.5). There was no significant main effect of STIMULATION CONDITION (*p* = 0.44) or any significant interaction effects involving STIMULATION CONDITION (Fig. 6*A*).

10.1523/ENEURO.0026-26.2026.f6-1 Figure 6-1 Simple reaction time results. Mean reaction time was unchanged from pre to post sham (left) and real rTMS (right), regardless as to which stimuli type was used to cue responses and whether left hand, right hand, or bimanual responses were required. Points represent group mean values and error bars represent 95% confidence intervals. Download Figure 6-1, TIF file.

#### SD of RT

SD of RT was greater when responses were cued with symbolic stimuli compared with spatial stimuli as was evidenced by a significant main effect of STIMULUS TYPE (*β* = 32; 95% CI [15 48]; *p* < 0.001; *d* = 1.06). This main effect should be considered in the context of a significant STIMULUS TYPE × RESPONSE TYPE interaction effect (*β* = 30; 95% CI [12 48]; *p* = 0.001). Estimated marginal means were calculated to further interrogate the interaction effect. Asymmetric responses were found to be significantly slower than symmetric responses when trials were cued with symbolic stimuli (*µ* = 33 ± 10; *p* = 0.01; *d* = 0.47) but not when cued with spatial stimuli (*µ* = 6 ± 10; *p* = 0.92; *d* = 0.15). There was no significant main effect of STIMULATION CONDITION (*p* = 0.83) or any significant interaction effects involving STIMULATION CONDITION (Fig. 6*B*).

### Simple RT task

Mean RT did not significantly differ across levels of STIMULATION CONDITION (*p* = 0.083), TIME (*p* = 0.13), RESPONSE TYPE (unimanual-right/unimanual-left, *p* = 0.5; unimanual-right/bimanual, *p* = 0.14), or STIMULUS TYPE (*p* = 0.11). Furthermore, there were no significant interaction effects (Extended Data Fig. 6-1).

### Corticospinal excitability

**Figure 7.**
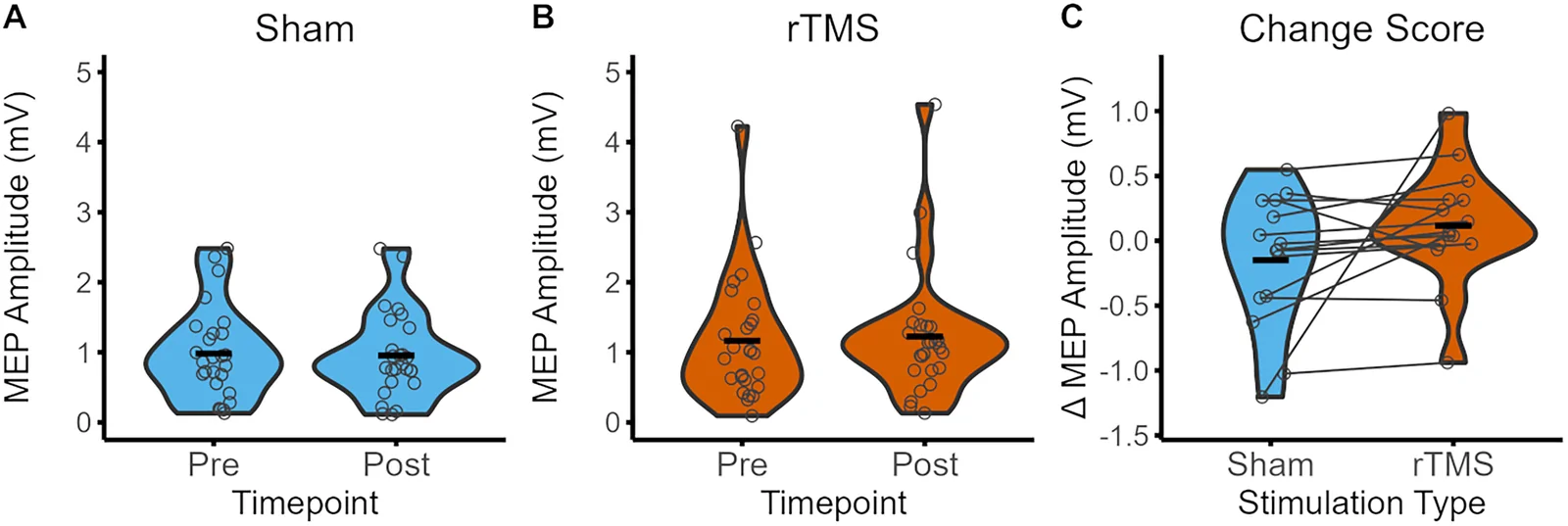
Corticospinal excitability across sham and real rTMS. Corticospinal excitability was unchanged from pre to post sham (***A***) and real rTMS (***B***). Horizontal black bars represent group mean. Circles represent individual participants. For change scores (***C***), lines link individual participants across stimulation conditions. Change scores are included to demonstrate that the vast majority of participants showed similar changes in corticospinal excitability across rTMS and sham stimulation.

Corticospinal excitability did not differ significantly across levels of TIME (*p* = 0.86) or STIMULTION CONDITION (*p* = 0.265), and there was no significant TIME × STIMULATION CONDITION interaction effect (*p* = 0.71; Fig. 7).

### Assessing the strength of the rTMS null effect with Bayesian statistics

Results from the exploratory Bayesian repeated-measures ANOVA on mean absolute error data mirrored those found for LMER analysis. Most evidence was found for the model including a main effect of STIMULUS TYPE, a main effect of RESPONSE TYPE, and an interaction effect of MOVEMENT FREQUENCY with RESPONSE TYPE and no effect of STIMULATION CONDITION. This model was found to have a Bayes factor of the alternative hypothesis over the null hypothesis (BF_10_) of 2.65 × 10^8^. The best fit model including a two-way interaction effect between STIMULATION CONDITION and RESPONSE TYPE (i.e., alternative Hypothesis 1 described in Fig. 1) had a BF_10_ equal to 1.773 × 10^7^, 15 times less than the best fit model which included no effect of STIMULATION CONDITION. Even weaker evidence was found for the best fit model including a three-way interaction between STIMULATION CONDITION, RESPONSE TYPE, and STIMULUS TYPE (i.e., alternative Hypothesis 2 described in Fig. 1). This model had a BF_10_ of 1.18 × 10^6^, which equated to 225 times less evidence than the best fit model with no effect of STIMULATION CONDITION.

## Discussion

In the current study, we found no effect of 1 Hz rTMS over right PMd on the stability of bimanual rhythmic finger tapping relative to a sham rTMS control condition. As expected, asymmetric movements were less stable than symmetric movements at high movement frequencies. However, when compared with sham stimulation, rTMS was not associated with a significant decrease in asymmetric movement stability relative to symmetric movement stability. Similarly, task conditions in which movement frequency was cued with abstract symbolic stimuli were not significantly less stable after rTMS compared with conditions in which movement frequency was cued with spatially congruent stimuli (Fig. 5). Importantly, this null effect of stimulation was unchanged in a follow-up analysis restricted to the 3 Hz condition (Extended Data Fig. 5-1), suggesting that the absence of a stimulation effect was not attributable to reduced sensitivity in the omnibus model arising from the large number of within-subject factors and interactions. This interpretation was further supported by a follow-up analysis of within-hand ITI variability restricted to the 3 Hz condition, which likewise showed greater variability during asymmetric/symbolic tapping but no evidence that rTMS selectively destabilized either hand relative to sham (Extended Data Fig. 5-2). Bimanual choice RT (Fig. 6), bimanual simple RT, and left and right hand unimanual simple RT (Extended Data Fig. 6-1) were also unchanged after rTMS relative to sham stimulation, regardless of the stimulus type used to cue movement or whether asymmetric or symmetric responses were required. Corticospinal excitability was not affected by rTMS or sham stimulation (Fig. 7) suggesting that any effects of the stimulation protocol were not associated with measurable spread to right M1 corticospinal circuits. Taken together, these findings indicate that 1 Hz rTMS over right PMd was not associated with a measurable disruption of performance relative to sham in the current paradigm. This suggests that no large or readily observable behavioral consequence of right PMd rTMS was detectable under the present stimulation and testing conditions. However, preserved performance following rTMS does not exclude the possibility that right PMd is functionally specialized for these processes under normal operating conditions; rather, it suggests that transient challenge to right PMd was not causally sufficient to disrupt performance in the current paradigm. Because we did not directly verify the degree of modulation of PMd with concurrent neural data, this interpretation should be made with appropriate caution.

### The brain may have the capacity to compensate for neuronal challenge to right PMd to maintain rhythmic bimanual behaviors

Previous neurophysiological evidence demonstrated a link between PMd activity and asymmetric bimanual rhythmic control (Meyer-Lindenberg et al., 2002; Wenderoth et al., 2005; Aramaki et al., 2006; van den Berg et al., 2010), while behavioral evidence has associated asymmetric bimanual control with an increased load on cognitive resources (Summers et al., 1998; Byblow et al., 1999; Mechsner et al., 2001; Wenderoth et al., 2009). Therefore, we predicted that detriments to the stability of temporal coupling of rhythmic bimanual movements after rTMS over right PMd would scale with the degree of cognitive control demands engendered by task conditions. Symmetric and asymmetric rhythmic tapping were assessed with either flickering spatially congruent visual stimuli or flickering abstract symbolic visual stimuli demonstrating the desired movement frequency in distinct blocks of rhythmic tapping. It has previously demonstrated that cueing movement frequency with spatially congruent stimuli significantly increases the stability of bimanual rhythmic tapping behaviors when compared with abstract symbolic stimuli, probably by reducing the strain on cognitive resources (Denyer and Boyd, 2025). Therefore, these task manipulations provided a method to effectively test whether right PMd was responsible for programming asymmetric rhythmic movements or more broadly responsible for managing increased cognitive control demands during rhythmic bimanual behaviors.

Rather than observing detriments to task performance that scaled with the degree of cognitive control demands or detriments to performance specifically in asymmetric tapping conditions, we found no change in task performance after 1 Hz rTMS over right PMd compared with sham stimulation. Of course, null effects should be interpreted with caution, as they may reflect insufficient statistical power, measurement error, or design limitations rather than the true absence of an effect (Lakens, 2017). Bayesian statistics can be useful for interpreting null effects (Quintana and Williams, 2018), and in this instance they indicated that a model with no effect of stimulation condition was supported 14 times more than a model with an interaction between stimulation condition and response type and 225 times more than a model with an interaction between stimulation condition, response type, and stimulus type. However, because the present study did not include a direct physiological measure confirming modulation of right PMd, these Bayesian results should be interpreted as strengthening the conclusion that no detectable behavioral effect of stimulation was observed in the current dataset rather than as evidence that right PMd makes no functional contribution to asymmetric bimanual control. While the lack of direct measurement of the status of right PMd following rTMS/sham limits interpretation, the present findings constrain strong claims that transient challenge to right PMd is sufficient to impair asymmetric rhythmic movements or the management of increased cognitive control demands in rhythmic bimanual movement contexts, as described in our competing hypotheses (Fig. 1), rather than excluding smaller or more subtle effects.

While it is important to bear in mind that the lack of direct measurement on right PMd post-rTMS constrains speculation, one possible explanation for the present findings is that right PMd forms one node within a broader cortical network supporting rhythmic bimanual behavior, allowing behavioral performance to be maintained despite transient challenge to right PMd. Existing evidence that the brain can compensate for neuronal challenge to PMd comes from a study which applied 1,200 pulses of 1 Hz rTMS over left PMd prior to the performance of unimanual decision-making task with the right hand (O'Shea et al., 2007a). This work found that choice RT was slowed immediately after rTMS over left PMd but returned to baseline levels ∼4 min after stimulation. Additionally, this rebound in choice RT was associated with an increase in blood-oxygen-dependent signal in a network of premotor regions beyond left PMd, including right PMd, right M1, left SMA, and bilateral CMA. This increase in activation suggests that a broader network of cortical regions was able to compensate for decreased excitability in left PMd to stabilize decision-making processes. Similarly, the application of short trains of five pulses of rTMS at 10 Hz over left PMd “online” during preparation of unimanual responses in a simple RT paradigm is shown to have no effect on RT (Duque et al., 2012). However, application of five pulses of rTMS at 10 Hz over PMd was shown to concurrently disrupt PMd–M1 interhemispheric circuits. Given that PMd–M1 interhemispheric circuits are thought to play a role in sculpting corticospinal output during unimanual preparation (Derosiere and Duque, 2020; Neige et al., 2021; Denyer et al., 2023), this further suggests that a broader network of regions can account for challenge to PMd to maintain behavioral output within certain behavioral paradigms.

Similar compensatory processes may have occurred in the current study. Studies using fMRI have demonstrated that a network of frontal and parietal regions including right PMd as well as the superior parietal cortex and the intraparietal cortex are activated during difficult bimanual rhythmic tasks that require continuous movement of two hands in asymmetric directions (Wenderoth et al., 2005). Similarly, asymmetric rhythmic bimanual finger tapping is associated with increases in activation in right PMd but also in the left supramarginal gyrus, bilateral SMA, and bilateral CMA (Meyer-Lindenberg et al., 2002; Aramaki et al., 2006). Therefore, given that a network of frontal–parietal cortical regions beyond right PMd is recruited during bimanual rhythmic behaviors, perhaps the application of 1 Hz rTMS over right PMd in the current study may have triggered an increase in activation in this network of cortical regions, mitigating any potential behavioral consequence of rTMS. The involvement of a larger network may have been masked in previous studies which found increased switching from asymmetric to symmetric bimanual tapping behaviors after application of single pulses of TMS over PMd (Meyer-Lindenberg et al., 2002; van den Berg et al., 2010), since a period of ∼4 min is needed for compensation processes to take effect (O'Shea et al., 2007a). This may have given the false impression that PMd is critical for asymmetric bimanual control, when in reality a larger cortical network with built-in redundancies that can compensate for challenge to PMd could be recruited during asymmetric bimanual behaviors. However, the preserved behavioral output observed in the current study could reflect either such network-level compensation or simple attenuation of inhibitory effects over the poststimulation testing interval. Because no concurrent neural measure of PMd engagement was obtained, the present design cannot fully distinguish between these possibilities.

### Limitations

The primary limitation of the current study is that no direct physiological measure was obtained to verify that 1 Hz rTMS effectively modulated right PMd. Consequently, the null behavioral findings cannot distinguish between a genuinely limited behavioral contribution of right PMd under the present task conditions and insufficient or variable modulation of the target region. While 1 Hz rTMS is understood to inhibit cortical activity in healthy participants, the effects can be variable between participants (Maeda et al., 2000; Ridding and Ziemann, 2010; Caparelli et al., 2012; Weisz et al., 2012; Goldsworthy et al., 2021). Furthermore, the time course of rTMS-induced effects on cortical inhibition differs between individuals (Peinemann et al., 2004). Such mixed effects of rTMS over PMd may have produced a confounding factor that added noise to behavioral measures. Because we did not collect concurrent measures of cortical activity, our statistical models were not able to account for this potential confounding factor. Furthermore, while we have discussed the likelihood that compensatory processes facilitated the maintenance of behavioral output despite inhibition of right PMd based on previous research, the lack of a concurrent neural measure means we have no evidence that such neural compensation actually occurred in the current study or what the spatial extent of compensation might have been. To effectively index cortical inhibition and account for potential compensatory processes, it is critical that future investigations concurrently collect neural measures before and after 1 Hz rTMS over PMd. Functional MRI is well suited is this regard since it has excellent spatial resolution and has previously been shown to able to capture neural compensation during decision-making after 1 Hz rTMS over PMd (O'Shea et al., 2007a). Advanced diagnostic TMS methods may also be useful for indexing the inhibitory and compensatory effects of rTMS. While we collected single-pulse TMS measures before and after rTMS in the current study, the purpose of this measure was to verify that stimulation over right PMd did not directly alter right M1 corticospinal excitability. As such, this measure does not provide a direct index of PMd target engagement. The application of dual-coil TMS to measure interhemispheric circuits between PMd and M1 may be better placed to directly capture reductions in PMd excitability after 1 Hz rTMS (Mochizuki et al., 2004). Similarly, dual-coil TMS could be used to measure changes in activity in other cortical regions hypothesized to produce compensatory activity after inhibition of PMd with rTMS, such as SMA (Arai et al., 2012), M1 (Daskalakis et al., 2002), and the posterior parietal cortex (Vesia et al., 2013, 2017). While PMd–M1 interhemispheric circuits may be less active during the preparation of bimanual movements (Denyer et al., 2024), they may become active when compensation is triggered by neuronal challenge to contralateral PMd.

Another possible limitation is that the effects of 1 Hz rTMS may have attenuated during the poststimulation interval. However, poststimulation testing began promptly, and all behavioral testing was completed within ∼25.5 min of stimulation offset (Table 2), making it unlikely that the null findings were due simply to excessive delay in behavioral assessment relative to rTMS. Nevertheless, because the magnitude and time course of 1 Hz rTMS effects can vary across individuals, some attenuation over time cannot be ruled out.

A related limitation is that condition order within the rhythmic tapping task was only partially counterbalanced. Although cue type order was counterbalanced, response type was fixed such that asymmetric conditions were always completed before symmetric conditions, and movement frequency always increased from 1.4 to 2.2 to 3 Hz. This design choice reduced the risk of participants beginning at the most difficult frequency and ensured that the asymmetric conditions were tested earlier in the poststimulation period, but it may also have introduced practice, fatigue, or adaptation effects that could have influenced behavioral performance. However, substantial fatigue effects are unlikely given that the rhythmic tapping task was completed within ∼13 min.

A further limitation is that we did not test different varieties of abstract cues in our choice RT task and bimanual rhythmic task. This is important, since the abstract cues that we did use were single words (IN, OUT, LEFT, RIGHT) that communicated a unified representation of the stimulus–response pair, which differed somewhat to the more independent nature of the four spatial stimuli deployed. The unified nature of the abstract cues we used means that the effects observed (or lack thereof) may have been partially driven by this difference as opposed to solely being driven by a spatial-abstract dichotomy. Indeed, previous work in bimanual finger circling (Wenderoth et al., 2009) and bimanual pointing (Stanciu et al., 2017) have shown that abstract cues with spatial elements (e.g., arrows pointing in the desired direction) can reduce asymmetric interference effects versus more traditional abstract cues such as singular letters. While time is always of the essence in rTMS paradigms, future studies could dive deeper on this question by testing additional classes of nonunified abstract stimuli, such as single letter cues (e.g., M for middle finger movement, I for index finger movement) and abstract stimuli with a spatial component (e.g., arrows).

### Conclusion

Results from the current study indicate that 1 Hz rTMS over right PMd did not induce a detectable effect on the stability of bimanual rhythmic tapping, on bimanual choice RT, or on simple RT, regardless of the perceptual quality of the stimulus used to cue movement timing or whether asymmetric or symmetric responses were required. These findings constrain claims that transient challenge to right PMd is sufficient to disrupt asymmetric movement programming or the management of cognitive control demands in the present task context. However, because no concurrent index of PMd status was obtained, the null result cannot distinguish between a genuinely limited behavioral consequence of right PMd perturbation and insufficient or variable engagement of the target by rTMS. While compensatory activity within a distributed motor network is a plausible account for the preserved performance, insufficient or variable target engagement remains an equally viable explanation for the lack of detectable behavioral effects. Future studies incorporating concurrent neural measures alongside bimanual behavioral paradigms can help to distinguish between these possibilities more directly.

## Synthesis

Reviewing Editor: David Franklin, Technische Universitat Munchen Decisions are customarily a result of the Reviewing Editor and the peer reviewers coming together and discussing their recommendations until a consensus is reached. When revisions are invited, a fact-based synthesis statement explaining their decision and outlining what is needed to prepare a revision will be listed below. The following reviewer(s) agreed to reveal their identity: Yu Aramaki.

Your manuscript has now been reviewed to two experts in the field. While they both appreciate the scientific contribution of this work, we all have concerns with the current version of the manuscript. In particular, I highlight our key concerns which need to be addressed: lack of direct validation of PMd modulation; timing of behavioral testing relative to rTMS; and the strength of the casual inference and interpretation of null effects. The lack of direct physiological validation of PMd is of particular concern, as it affects how we can interpret the null effects. In addition, we think that a more focussed analysis of the 3Hz asymmetric condition and analyses of hand-specific variability would be helpful. I have combined both reviews comments and suggestions into a single review below. As they both highlight some of the same issues, some of the comments partially overlap. Please revise the manuscript using these comments and suggestions.

Specific comments from the reviewers

The study addresses an important and longstanding question regarding the role of the dorsal premotor cortex in asymmetric bimanual coordination and contributes useful empirical data, particularly given the relative scarcity of well-controlled null-result TMS studies in this domain. This manuscript examines whether inhibitory rTMS applied to the right dorsal premotor cortex (PMd) influences bimanual coordination and reaction time performance under varying response symmetry (symmetric vs. asymmetric) and stimulus type (spatial vs. symbolic). Using a within-subject design with a sham control, the authors report clear behavioral effects of task demands but no detectable impact of rTMS on performance or corticospinal excitability. The study is well motivated, methodologically organized, and addresses an important question regarding PMd contributions to motor coordination and cognitive load. However, the central conclusions currently extend beyond what the data can support. In particular, the absence of a convincing manipulation check for effective PMd modulation, along with limited constraints on null effect interpretation, weakens the strength of the mechanistic claims.

Major Comments

The timing of behavioral tasks following rTMS requires clarification. The experimental sequence (MEP assessment, followed by simple reaction time, rhythmic tapping, and then choice reaction time) introduces a delay between stimulation and the primary behavioral outcomes, yet this delay is not quantified. Given that the effects of 1 Hz rTMS are time-dependent and may diminish or be compensated for within minutes, this omission limits interpretability. Without explicit timing information, it is difficult to determine whether the null results reflect a true absence of effect or a reduction of stimulation effects due to temporal delay. Reporting the approximate time between the final rTMS pulse and the onset of each behavioral task (including variability across participants), clarifying whether timing was standardized, and discussing how this delay may influence sensitivity to detect behavioral effects would strengthen the manuscript.

Temporal Window of Testing: "Compensation" vs. "Physiological Decay"

The authors cite O'Shea et al. (2007) to suggest that network-level reorganization can occur within minutes following rTMS. However, the procedure included MEP assessments and a simple RT task before the main bimanual rhythmic tapping task. The authors should provide a precise timeline (in minutes) from the end of rTMS to the start of the rhythmic tapping task, and specifically to the onset of the 3 Hz asymmetric condition. Given that the behavioral and physiological effects of 1 Hz rTMS can be transient, the null effect could reflect not only network-level compensation but also a simple decay of the inhibitory effect during the post-rTMS interval. If the high-load conditions were tested after the effective window had partially or fully dissipated, the null result may largely reflect experimental timing limitations rather than providing strong evidence regarding network architecture or node specialization.

The manuscript makes relatively strong causal inferences about the role of right PMd despite lacking clear validation that the stimulation protocol effectively modulated the targeted region. The null behavioral effects are interpreted as evidence against a specialized role of PMd in asymmetric coordination and cognitive load. However, there is no direct confirmation that PMd activity was altered. The only physiological measure provided (MEPs from M1) is indirect and did not show a significant effect, further limiting interpretability. As such, it remains unclear whether the null behavioral findings reflect a lack of PMd involvement or insufficient modulation of the target. The conclusions would benefit from reframing to emphasize that no detectable behavioral effect was observed under the present stimulation conditions, rather than ruling out PMd function. It would also be important to more explicitly acknowledge limitations related to target engagement and distinguish between absence of evidence and evidence of absence.

Lack of Physiological Confirmation and Bayesian Interpretation. While the authors confirmed that right M1 excitability was unchanged, there was no direct physiological measure (e.g., TMS-EEG, dual-coil TMS, or connectivity-based TMS measures) to verify that right PMd activity was actually suppressed. This is a critical limitation when interpreting Bayesian support for a null effect. A Bayes Factor supporting the null hypothesis is only meaningful if the experimental manipulation successfully modulated the intended neural target. Without evidence of target engagement, the null effect might reflect insufficient modulation of the PMd rather than true functional redundancy. This concern is distinct from the timing issue (potential decay). Even if the inhibitory effect persisted throughout testing, the absence of a direct PMd engagement measure limits inference about PMd's causal contribution.

Distinction Between Functional Specialization and Causal Necessity. The manuscript interprets preserved behavioral performance after rTMS as evidence that the right PMd is not a specialized control center. However, there is a crucial conceptual distinction between causal necessity and functional specialization. The current results suggest that the right PMd may not be causally necessary to maintain performance under the specific experimental conditions tested, potentially due to redundancy or resilience within the bimanual control network. This does not, however, exclude the possibility that the node is functionally specialized for such processes under normal, non-perturbed operating conditions. I therefore recommend moderating the claims and reframing the results primarily in terms of network robustness rather than absence of specialization.

Sensitivity of Relative Phase vs. Hand-Specific Variability. Relative phase is a standard and appropriate measure of interlimb coupling. However, it can mask unilateral destabilization effects. For example, if the dominant hand strongly entrains the non-dominant hand, overall coupling may appear stable even if control of one hand is degraded. Prior literature suggests that the right PMd may contribute to stabilizing the non-dominant hand during asymmetric coordination. I strongly recommend reporting hand-specific timing variability (e.g., inter-tap interval variability for each hand separately). This would clarify whether subtle unilateral effects were present but not detectable using relative phase alone.

Focused Analysis of the High-Demand (3 Hz) Condition. The behavioral data indicate that asymmetric movements only became significantly unstable at 3 Hz. This suggests that PMd contribution may become critical only when the motor system approaches performance limits.

By collapsing data across frequencies in several analyses and visualizations, potential stimulation effects specific to the highest-demand condition may be diluted. A focused analysis of the 3 Hz asymmetric condition would provide a more sensitive test of potential rTMS effects.

The manuscript would benefit from stronger integration with established frameworks in bimanual coordination, particularly those related to coordination stability, symmetry constraints, and neural crosstalk. A substantial body of work shows that symmetric (homologous) movements are more stable and less computationally demanding than asymmetric (non-homologous) movements, with asymmetry costs often attributed to interference from neural crosstalk or increased control demands to suppress it. While the present study leverages symmetric versus asymmetric conditions, these theoretical frameworks are not clearly incorporated into the hypotheses or interpretation. The observed performance differences, as well as their modulation by movement frequency, could be interpreted in terms of stability constraints, interference dynamics, or limitations in maintaining independent limb control at higher frequencies. Integrating these perspectives would strengthen the theoretical grounding and help clarify whether the null effects of rTMS reflect distributed redundancy, insufficient perturbation, or task dynamics dominated by well-established coordination constraints.

The interpretation of the null effects would benefit from more explicit constraints on statistical power and detectable effect sizes. While Bayesian analyses are included, the manuscript does not clearly specify what magnitude of effect the study was adequately powered to detect. The design appears primarily powered for large effects, whereas smaller or moderate effects of rTMS are both plausible and commonly observed, particularly given known variability in behavioral responses to stimulation. Without defining these limits, the conclusions risk overstating what can be inferred from the null findings. Clarifying the range of detectable effect sizes and stating that the results primarily rule out large effects, rather than more subtle influences, would provide a more appropriately bounded interpretation.

Movement frequency clearly influences behavioral performance and appears to amplify asymmetry costs, yet it is not incorporated into the conceptual framework or predictions presented in the Introduction or Figure 1. As currently presented, frequency functions more as a statistical factor than a theoretically motivated variable. This raises questions about whether frequency is intended to reflect task difficulty, motor instability, or cognitive load, and whether it is expected to interact with response type in meaningful ways. Clarifying the intended role of frequency (e.g., control variable, theoretically relevant manipulation) would improve interpretability. If it is conceptually important, it should be incorporated into the hypotheses. If not, its role should be explicitly justified.

Clarification of task and condition order is needed. In repeated measures motor tasks, practice, fatigue, or adaptation effects can meaningfully influence performance and may obscure subtle effects of stimulation, particularly in a design intended to detect potentially small neuromodulatory effects. It would be helpful to specify whether task components and conditions were fully counterbalanced across participants and, if not, to justify this choice or consider accounting for order effects analytically.

The interpretation of symbolic versus spatial cues as a manipulation of cognitive load may be somewhat narrow. While symbolic cues likely increase cognitive demands, they also differ from spatial cues in abstraction, mapping requirements, and perceptual processing. As a result, it is difficult to attribute observed differences solely to cognitive load. The manuscript would benefit from a more cautious interpretation of this manipulation and earlier acknowledgment of alternative explanations, rather than limiting this discussion to the limitations section.

Minor Comments

1. Clarify how the FDI "hotspot" was identified and how motor threshold was determined.

2. Provide more detail on inclusion/exclusion criteria and handling of incorrect or missing response blocks across conditions.

3. Consider including a schematic for the simple reaction time task comparable to the bimanual mapping figure.

4. Some figures with multiple conditions are difficult to interpret. Simplifying these or moving detailed breakdowns to supplementary material would improve clarity.

5. Visualizing key results as a function of movement frequency may aid interpretation.

6. Provide clearer model specifications (fixed and random effects).

7. Consider reporting effect sizes alongside statistical tests.

8. Ensure consistent terminology when referring to stimulation effects (e.g., avoid implying confirmed "disruption" without direct evidence).

9. Consider visualizing key results as a function of movement frequency.:

10. Correct typographical errors (e.g., "cogntive").

## Author Response

Synthesis Statement for Author (Required):

Your manuscript has now been reviewed to two experts in the field. While they both appreciate the scientific contribution of this work, we all have concerns with the current version of the manuscript. In particular, I highlight our key concerns which need to be addressed: lack of direct validation of PMd modulation; timing of behavioral testing relative to rTMS; and the strength of the casual inference and interpretation of null effects. The lack of direct physiological validation of PMd is of particular concern, as it affects how we can interpret the null effects. In addition, we think that a more focussed analysis of the 3Hz asymmetric condition and analyses of hand- specific variability would be helpful. I have combined both reviews comments and suggestions into a single review below. As they both highlight some of the same issues, some of the comments partially overlap. Please revise the manuscript using these comments and suggestions. Specific comments from the reviewers The study addresses an important and longstanding question regarding the role of the dorsal premotor cortex in asymmetric bimanual coordination and contributes useful empirical data, particularly given the relative scarcity of well-controlled null-result TMS studies in this domain. This manuscript examines whether inhibitory rTMS applied to the right dorsal premotor cortex (PMd) influences bimanual coordination and reaction time performance under varying response symmetry (symmetric vs. asymmetric) and stimulus type (spatial vs. symbolic). Using a within- subject design with a sham control, the authors report clear behavioral effects of task demands but no detectable impact of rTMS on performance or corticospinal excitability. The study is well motivated, methodologically organized, and addresses an important question regarding PMd contributions to motor coordination and cognitive load. However, the central conclusions currently extend beyond what the data can support. In particular, the absence of a convincing manipulation check for effective PMd modulation, along with limited constraints on null effect interpretation, weakens the strength of the mechanistic claims.

We thank the editor and reviewers for the time and care taken in evaluating our manuscript. We particularly appreciate the effort to integrate the reviews into a single coherent set of comments, which made the revision process more streamlined. We found the feedback fair and constructive, and have made substantial changes to the manuscript in response. We believe these revisions have significantly strengthened the quality and clarity of the work. In this document, we provide a point-by-point response to each comment, together with a description of the corresponding changes made to the manuscript. All changes in the revised manuscript are highlighted in red. Major Comments The timing of behavioral tasks following rTMS requires clarification. The experimental sequence (MEP assessment, followed by simple reaction time, rhythmic tapping, and then choice reaction time) introduces a delay between stimulation and the primary behavioral outcomes, yet this delay is not quantified. Given that the effects of 1 Hz rTMS are time-dependent and may diminish or be compensated for within minutes, this omission limits interpretability. Without explicit timing information, it is difficult to determine whether the null results reflect a true absence of effect or a reduction of stimulation effects due to temporal delay. Reporting the approximate time between the final rTMS pulse and the onset of each behavioral task (including variability across participants), clarifying whether timing was standardized, and discussing how this delay may influence sensitivity to detect behavioral effects would strengthen the manuscript.

We appreciate this point and agree that the timing of post-stimulation testing should be reported explicitly. We have now quantified the interval between the end of stimulation and the onset of each post-stimulation task and added these values to the manuscript. The first post-stimulation assessment began within approximately 2 min of stimulation offset, reflecting the time required to collect 10 post-stimulation MEPs at 5 s intervals and to adjust the setup before the simple RT task. Mean task-onset latencies were similar between sessions: simple RT began at 2.0 min, rhythmic bimanual tapping at 5.0 min, and bimanual choice RT at 12.9 min after rTMS, compared with 2.0, 5.0, and 13.1 min after sham stimulation, respectively. Total post-stimulation testing was completed within 25.5 min in all sessions. We have also revised the Discussion to clarify that, while these timings make it unlikely that the null findings were due simply to excessive delay in behavioral assessment, attenuation of 1 Hz rTMS effects over time cannot be fully excluded.

In response to this comment, we added a new Table 2 which contains details of the precise timing of all assessments post rTMS/Sham.

We also added the following text to the end of the Procedure section in Materials and Methods: "To clarify the precise timing of post-stimulation behavioural testing, we quantified the interval between the end of stimulation and the onset of each post-stimulation task. The first post- stimulation assessment began within approximately 2 min of stimulation offset, reflecting the time required to collect 10 post-stimulation MEPs at 5 s intervals and to adjust the setup before the simple RT task. Mean task-onset latencies were similar between sessions: simple RT began at 2.0 min, rhythmic bimanual tapping at 5.0 min, and bimanual choice RT at 12.9 min after rTMS, compared with 2.0, 5.0, and 13.1 min after sham stimulation, respectively. Timing was similar between sessions and differed little between participants (Table 2)." We also added the following text to the limitations section in the Discussion: "Another possible limitation is that the effects of 1 Hz rTMS may have attenuated during the post-stimulation interval. However, post-stimulation testing began promptly, and all behavioral testing was completed within ~25.5 minutes of stimulation offset (Table 2), making it unlikely that the null findings were due simply to excessive delay in behavioral assessment relative to rTMS. Nevertheless, because the magnitude and time course of 1 Hz rTMS effects can vary across individuals, some attenuation over time cannot be ruled out." Temporal Window of Testing: "Compensation" vs. "Physiological Decay" The authors cite O'Shea et al. (2007) to suggest that network-level reorganization can occur within minutes following rTMS. However, the procedure included MEP assessments and a simple RT task before the main bimanual rhythmic tapping task. The authors should provide a precise timeline (in minutes) from the end of rTMS to the start of the rhythmic tapping task, and specifically to the onset of the 3 Hz asymmetric condition. Given that the behavioral and physiological effects of 1 Hz rTMS can be transient, the null effect could reflect not only network-level compensation but also a simple decay of the inhibitory effect during the post- rTMS interval. If the high-load conditions were tested after the effective window had partially or fully dissipated, the null result may largely reflect experimental timing limitations rather than providing strong evidence regarding network architecture or node specialization.

To address this comment, we have now added explicit timing information for the post- stimulation testing sequence in the Methods and summarized these values in Table 2. Post- stimulation testing began within approximately 2 min of stimulation offset, the rhythmic tapping task began at approximately 5 min after stimulation, and all behavioral testing was completed within ~25.5 min. We also clarified the ordering of the rhythmic tapping conditions: asymmetric conditions were completed before symmetric conditions, and movement frequency increased from 1.4 to 2.2 to 3 Hz within each condition. Together, these additions allow the timing of the highest-demand conditions relative to stimulation offset to be inferred directly from the manuscript.

We agree that the null effect could reflect either network-level compensation or attenuation of inhibitory effects over time. Therefore, we have revised the Discussion to clarify that the present design cannot fully distinguish between these possibilities. At the same time, the timing information reported in Table 2 makes it unlikely that the null findings can be attributed simply to gross delay in post-stimulation testing, given that all rhythmic tapping blocks were completed well within the time range of effects typically accepted for 1 hertz rTMS and the period of neural compensation observed by O'Shea et al., (2007).

In response to this comment, we have added the following text to the Discussion just prior to the Limitations section: "However, the preserved behavioural output observed in the current study could reflect either such network-level compensation or simple attenuation of inhibitory effects over the post- stimulation testing interval. Because no concurrent neural measure of PMd engagement was obtained, the present design cannot fully distinguish between these possibilities." The manuscript makes relatively strong causal inferences about the role of right PMd despite lacking clear validation that the stimulation protocol effectively modulated the targeted region. The null behavioral effects are interpreted as evidence against a specialized role of PMd in asymmetric coordination and cognitive load. However, there is no direct confirmation that PMd activity was altered. The only physiological measure provided (MEPs from M1) is indirect and did not show a significant effect, further limiting interpretability. As such, it remains unclear whether the null behavioral findings reflect a lack of PMd involvement or insufficient modulation of the target. The conclusions would benefit from reframing to emphasize that no detectable behavioral effect was observed under the present stimulation conditions, rather than ruling out PMd function. It would also be important to more explicitly acknowledge limitations related to target engagement and distinguish between absence of evidence and evidence of absence.

We appreciate this important point and upon reflection agree that the original manuscript drew stronger causal inferences than are warranted in the absence of a direct physiological measure confirming modulation of right PMd. In the revised manuscript, we have therefore reframed our interpretation throughout to emphasize that we observed no detectable behavioral effect of 1 Hz rTMS over right PMd under the present stimulation and testing conditions, rather than interpreting the null result as evidence that right PMd plays no functional role in asymmetric coordination or in the management of cognitive load.

We now state more explicitly that the corticospinal excitability measure was included to assess whether stimulation effects spread to right M1, but does not provide a direct index of PMd target engagement. Accordingly, the null behavioral findings cannot distinguish between several possibilities, including insufficient or variable modulation of PMd, preserved behavioral output despite successful PMd perturbation, or a genuinely limited behavioral consequence of transient right PMd challenge under the present task conditions. We have revised the Abstract, Significance Statement, Discussion, Limitations, and Conclusion to reflect this distinction more clearly and to avoid conflating absence of evidence with evidence of absence.

In response to this comment, we have made the following specific changes to the manuscript. First, we have changed the end of the Abstract to make a less strongly worded claim: "Contrary to expectations, hypothesized rTMS-related alterations in tapping stability and accuracy were not dependent on movement symmetry or the degree of cognitive control demands. Instead, no detectable behavioral effect of rTMS was observed. These results indicate that asymmetric rhythmic tapping can be maintained under the present right PMd rTMS protocol and task conditions. Further work incorporating concurrent neural measures is needed to determine the precise neural mechanism underlying this null effect." We also updated the Significance Statement to be more cautious in terms of the claims made: "How the brain coordinates complex, two-handed movements remains a fundamental question in neuroscience with important clinical consequences. The current study tests whether the right dorsal premotor cortex (PMd) is specifically responsible for programming asymmetric movements or if it more broadly manages the increased cognitive control demands inherent to such movements. By using non-invasive brain stimulation to transiently challenge right PMd during bimanual movements, we found that bimanual stability remained unaffected regardless of movement symmetry or cognitive control demands condition. These findings indicate that, under the present stimulation and testing conditions, transient challenge to right PMd was not sufficient to measurably disrupt performance. The results therefore highlight the resilience of bimanual coordination while also emphasizing the need for concurrent neural measures when interpreting null effects of rTMS on complex behaviour." Within this Discussion we made several changes. The end of the first paragraph now reads: "Taken together, these findings indicate that 1 Hz rTMS over right PMd was not associated with a measurable disruption of performance relative to sham in the current paradigm. This argues against a large and readily observable behavioral consequence of transient right PMd challenge under the present conditions. However, preserved performance following rTMS does not exclude the possibility that right PMd is functionally specialized for these processes under normal operating conditions; rather, it suggests that transient challenge to right PMd was not causally sufficient to disrupt performance in the current paradigm. Because we did not directly verify the degree of modulation of PMd with concurrent neural data, this interpretation should be made with appropriate caution.

The part discussing the Bayesian analysis in the Discussion has been updated to read as follows: "However, because the present study did not include a direct physiological measure confirming modulation of right PMd, these Bayesian results should be interpreted as strengthening the conclusion that no detectable behavioral effect of stimulation was observed in the current dataset, rather than as evidence that right PMd makes no functional contribution to asymmetric bimanual control. While the lack of direct measurement of the status of right PMd following rTMS/sham limits interpretation, the present findings constrain strong claims that transient disruption of right PMd is sufficient to impair asymmetric rhythmic movements or the management of increased cognitive load in rhythmic bimanual movement contexts, as described in our competing hypotheses (Figure 1), rather than excluding smaller or more subtle effects." The end of that same section where the possibility of compensation was also changed to a more cautious interpretation: "However, the preserved behavioural output observed in the current study could reflect either such network-level compensation or simple attenuation of inhibitory effects over the post- stimulation testing interval. Because no concurrent neural measure of PMd engagement was obtained, the present design cannot fully distinguish between these possibilities." In the Limitations section, while we had already discussed at length the problem with having no concurrent neural index of right PMd, we updated the opening sentence to the following: "The primary limitation of the current study is that no direct physiological measure was obtained to verify that 1 Hz rTMS effectively modulated right PMd. Consequently, the null behavioral findings cannot distinguish between a genuinely limited behavioral contribution of right PMd under the present task conditions and insufficient or variable modulation of the target region." We also adjusted the previous sentence about the lack of utility of single pulse TMS to detect status of right PMd to be more explicit: "While we collected single-pulse TMS measures before and after rTMS in the current study, the purpose of this measure was to verify that stimulation over right PMd did not directly alter right M1 corticospinal excitability. As such, this measure does not provide a direct index of PMd target engagement." Finally, we updated the entire conclusion section to have a more cautious tone: "Results from the current study indicate that 1 Hz rTMS over right PMd did not induce a detectable effect on the stability of bimanual rhythmic tapping, on bimanual choice RT, or on simple RT, regardless of the perceptual quality of the stimulus used to cue movement timing or whether asymmetric or symmetric responses were required. These findings constrain claims that transient challenge to right PMd is sufficient to disrupt asymmetric movement programming or the management of cognitive control demands in the present task context. However, because no concurrent index of PMd status was obtained, the null result cannot distinguish between a genuinely limited behavioral consequence of right PMd perturbation and insufficient or variable engagement of the target by rTMS. In light of previous research, one possible interpretation is that compensatory activity in a broader cortical network helped maintain behavioral output despite the rTMS challenge. Future studies incorporating concurrent neural measures alongside bimanual behavioral paradigms can help to distinguish between these possibilities more directly." Lack of Physiological Confirmation and Bayesian Interpretation. While the authors confirmed that right M1 excitability was unchanged, there was no direct physiological measure (e.g., TMS- EEG, dual-coil TMS, or connectivity-based TMS measures) to verify that right PMd activity was actually suppressed. This is a critical limitation when interpreting Bayesian support for a null effect. A Bayes Factor supporting the null hypothesis is only meaningful if the experimental manipulation successfully modulated the intended neural target. Without evidence of target engagement, the null effect might reflect insufficient modulation of the PMd rather than true functional redundancy. This concern is distinct from the timing issue (potential decay). Even if the inhibitory effect persisted throughout testing, the absence of a direct PMd engagement measure limits inference about PMd's causal contribution.

We agree with this point and have addressed it in the revised manuscript. Specifically, we now make clear that the absence of a direct physiological measure of PMd engagement is a critical limitation when interpreting the Bayesian support for a null behavioral effect. The M1 corticospinal excitability measure was included to assess whether stimulation effects spread to right M1, but it does not provide a direct index of whether right PMd was successfully factors strengthen the conclusion that no detectable behavioral effect of stimulation was observed in the present dataset, rather than providing evidence that right PMd makes no causal or functional contribution to asymmetric bimanual control. We also revised the Limitations section to state more explicitly that, without direct confirmation of PMd target engagement, the null behavioral findings cannot distinguish between insufficient or variable modulation of PMd and genuinely limited behavioral consequences of transient right PMd perturbation. We do not explicitly link the exact changes here, since they are covered in the response to the previous comment.

Distinction Between Functional Specialization and Causal Necessity. The manuscript interprets preserved behavioral performance after rTMS as evidence that the right PMd is not a specialized control center. However, there is a crucial conceptual distinction between causal necessity and functional specialization. The current results suggest that the right PMd may not be causally necessary to maintain performance under the specific experimental conditions tested, potentially due to redundancy or resilience within the bimanual control network. This does not, however, exclude the possibility that the node is functionally specialized for such processes under normal, non-perturbed operating conditions. I therefore recommend moderating the claims and reframing the results primarily in terms of network robustness rather than absence of specialization.

We appreciate this conceptual distinction and agree that the original manuscript did not draw it clearly enough. In the revised manuscript, we have moderated our claims to distinguish more explicitly between causal necessity and functional specialization. Specifically, we now frame the preserved behavioral performance after rTMS as indicating that transient challenge to right PMd was not causally sufficient to impair performance under the present stimulation and testing conditions, rather than as evidence that right PMd is not functionally specialized under normal operating conditions. This distinction is now made explicitly in the first paragraph of the Discussion, in the Abstract, and in the Significance Statement. The broader framing of the manuscript has also been revised accordingly. Below we do not detail all specific changes as most have been covered in our previous responses. However, we note the following specific change in the end of the first paragraph of the Discussion, which now reads as follows: "However, preserved performance following rTMS does not exclude the possibility that right PMd is functionally specialized for these processes under normal operating conditions; rather, it suggests that transient challenge to right PMd was not causally sufficient to disrupt performance in the present paradigm. Because we did not directly verify modulation of PMd with concurrent neural data, this interpretation should be made with appropriate caution." Sensitivity of Relative Phase vs. Hand-Specific Variability. Relative phase is a standard and appropriate measure of interlimb coupling. However, it can mask unilateral destabilization effects. For example, if the dominant hand strongly entrains the non-dominant hand, overall the right PMd may contribute to stabilizing the non-dominant hand during asymmetric coordination. I strongly recommend reporting hand-specific timing variability (e.g., inter-tap interval variability for each hand separately). This would clarify whether subtle unilateral effects were present but not detectable using relative phase alone.

To address the possibility that relative phase may have obscured unilateral timing disturbances, we conducted an additional analysis of hand-specific inter-tap interval (ITI) variability in the highest-demand condition (3 Hz), where any such effect should have been most apparent. For each finger and each block, we calculated the standard deviation of the ITI, normalized it to the ideal ITI for that block, and then averaged normalized values across fingers within each hand to yield separate within-hand variability measures for the left and right hands. These data were analyzed using a linear mixed-effects model with HAND, STIMULUS TYPE, RESPONSE TYPE, and STIMULATION CONDITION as fixed effects and participant as a random effect. This follow-up showed a significant effect of RESPONSE TYPE, indicating greater ITI variability during asymmetric than symmetric tapping, but no main effect of STIMULATION CONDITION, no HAND × STIMULATION CONDITION interaction, and no higher-order interaction involving HAND and STIMULATION CONDITION. Thus, we found no evidence that real stimulation selectively destabilized one hand in a way that was masked by the relative- phase measure.

In response to this comment, we have made the following additions to the manuscript. First in the Data Processing section of Materials and Methods we state: "To facilitate a supplementary analysis of the effects of rTMS on within hand coordination stability, within-hand inter-tap interval (ITI) variability was quantified from the key press timing values for the 3 Hz rhythmic tapping blocks. For each finger and each block, the standard deviation of the ITI was calculated and normalized to the ideal ITI for that block. Normalized ITI variability values were then averaged across fingers within each hand, yielding separate within- hand variability measures for the left and right hands." We also now state the following in the Dependant Variables section of Materials and Methods: "Intertap interval variability - The SD of intertap intervals was calculated for each finger, normalized to the ideal intertap interval for that block, and averaged across fingers within each hand to provide a measure of within-hand intertap interval variability." We also added the following text to the Supplementary Analyses subsection of the Statisical Analysis section in Materials and Methods: "4-finger bimanual rhythmic tapping task To determine whether relative phase may mask effects of rTMS on unilateral destabilization in the highest-demand movement frequency condition (3 Hz), an additional LMER was performed on normalized within-hand ITI variability from the 3 Hz blocks only. Fixed effects were HAND (left/right), STIMULUS TYPE (symbolic/spatial), RESPONSE TYPE (symmetric/asymmetric), and STIMULATION CONDITION (rTMS/sham), with Participant ID included as a random effect." We also added the following section to the Results: "Intertap interval variability - The analysis of normalized intertap interval SD data revealed a significant main effect of RESPONSE TYPE, indicating greater within-hand ITI variability during asymmetric than symmetric tapping (β = 0.08, 95% CI [0.02, 0.14], p = 0.009). A significant CUE CONDITION × RESPONSE TYPE interaction was also observed (β = 0.11, 95% CI [0.02, 0.19], p = 0.017). Estimated marginal means indicated that symbolic cues significantly increased intertap interval variability when asymmetric responses were required (µ = 0.09 {plus minus} 0.02, p = 0.0001, d = 0.86), but not when symmetric responses were required (µ = 0.02 {plus minus} 0.02, p = 0.34, d = 0.25). Notably, there was no main effect of STIMULATION CONDITION (p = 0.797), no HAND × STIMULATION CONDITION interaction (p = 0.797), and no higher- order interaction involving HAND and STIMULATION CONDITION (all p {greater than or equal to} 0.476). These results indicate that, although within-hand timing variability increased in the highest-demand tapping conditions, rTMS did not selectively destabilize either hand relative to sham stimulation (Supplementary Figure 3)." We also added a new Supplementary Figure 3 showcasing these results. Finally, we added the following text to the first paragraph of the Discussion: "This interpretation was further supported by a follow-up analysis of within-hand intertap interval variability restricted to the 3 Hz condition, which likewise showed greater variability during asymmetric/symbolic tapping but no evidence that rTMS selectively destabilized either hand relative to sham (Supplementary Figure 3)." Focused Analysis of the High-Demand (3 Hz) Condition. The behavioral data indicate that asymmetric movements only became significantly unstable at 3 Hz. This suggests that PMd contribution may become critical only when the motor system approaches performance limits.

By collapsing data across frequencies in several analyses and visualizations, potential stimulation effects specific to the highest-demand condition may be diluted. A focused analysis of the 3 Hz asymmetric condition would provide a more sensitive test of potential rTMS effects.

We appreciate this suggestion from the reviewers. Movement frequency was already included in the primary mixed-effects model, such that a robust stimulation effect specific to the highest- demand condition would be expected to emerge as an interaction involving STIMULATION CONDITION and MOVEMENT FREQUENCY. Nevertheless, to directly address the reviewer's concern, we conducted a focused follow-up analysis restricted to the 3 Hz blocks, where asymmetric coordination costs were greatest. This model retained the same general structure as the primary analysis, with RESPONSE TYPE, STIMULUS TYPE, and STIMULATION CONDITION entered as fixed effects and participant as a random effect. This follow-up likewise showed no evidence of a stimulation effect: there was no main effect of STIMULATION CONDITION and no interaction involving STIMULATION CONDITION. Thus, the null stimulation result was preserved even in the highest-demand condition. We have added this analysis to the manuscript and now include a supplementary figure showing the full 3 Hz breakdown across task conditions.

In response to this comment, we have made the following changes to the manuscript. First, we added the following paragraph to the Supplementary Analyses section with Statistical Analysis in Materials and Methods: "To provide a more focused test of stimulation effects in the highest-demand movement frequency condition, an additional LMER was performed on mean absolute error data from the 3 Hz blocks only. This follow-up was conducted because the primary omnibus model contained multiple within-subject factors and higher-order interactions, which may have reduced sensitivity to a stimulation effect specific to the highest-demand condition. Fixed effects were RESPONSE TYPE (symmetric/asymmetric), STIMULUS TYPE (symbolic/spatial), and STIMULATION CONDITION (rTMS/sham), with Participant ID included as a random intercept." Next, we added the following to the results section: "Because asymmetric tapping costs were greatest at 3 Hz, we conducted a follow-up analysis restricted to the 3 Hz blocks. This focused model revealed no main effect of STIMULATION CONDITION (β = -0.04, 95% CI [-2.02, 1.95], p = 0.970), no RESPONSE TYPE × STIMULATION CONDITION interaction (p = 0.878), no STIMULUS TYPE × STIMULATION CONDITION interaction (p = 0.751), and no RESPONSE TYPE × STIMULUS TYPE × STIMULATION CONDITION interaction (p = 0.780). Thus, even in the highest-demand movement frequency condition, there was no evidence that rTMS stimulation impaired performance relative to sham (Supplementary Figure 2)." Finally, we added the following to the first paragraph of the Discussion: "Importantly, this null effect of stimulation was unchanged in a follow-up analysis restricted to the 3 Hz condition (Supplementary Figure 2), suggesting that the absence of a stimulation effect was not attributable to reduced sensitivity in the omnibus model arising from the large number of within-subject factors and interactions." The manuscript would benefit from stronger integration with established frameworks in bimanual coordination, particularly those related to coordination stability, symmetry constraints, and neural crosstalk. A substantial body of work shows that symmetric (homologous) movements are more stable and less computationally demanding than asymmetric (non-homologous) movements, with asymmetry costs often attributed to interference from neural crosstalk or increased control demands to suppress it. While the present study leverages symmetric versus asymmetric conditions, these theoretical frameworks are not clearly incorporated into the hypotheses or interpretation. The observed performance differences, as well as their modulation by movement frequency, could be interpreted in terms of stability constraints, interference dynamics, or limitations in maintaining independent limb control at higher frequencies. Integrating these perspectives would strengthen the theoretical grounding and help clarify whether the null effects of rTMS reflect distributed redundancy, insufficient perturbation, or task dynamics dominated by well-established coordination constraints.

We agree that some more time could be paid to existing theoretical accounts. Therefore, we have altered the Introduction to situate the task within established frameworks of bimanual coordination, including coordination stability, symmetry constraints, and interference/crosstalk between the two hands. We also clarify the role of movement frequency as an additional task- demand manipulation expected to amplify asymmetry-related costs. We did not substantially expand the Discussion on this point, as these frameworks primarily serve to better motivate the task design and interpretation of the observed asymmetry and frequency effects.

In response to this comment, we added the following section to the Introduction after the 2nd paragraph: "More broadly, the rhythmic tapping tasks also sit within established frameworks of bimanual coordination emphasizing stability constraints and symmetry biases. Symmetric bimanual movements are generally more stable than asymmetric movements, particularly as movement frequency increases, and asymmetry-related performance costs have often been interpreted in terms of neural crosstalk or increased control demands required to suppress interference between the two hands and maintain independent limb actions (Byblow et al., 1999; Mechsner et al., 2001; Summers et al., 1998; Temprado et al., 1999; Wenderoth et al., 2009). From this perspective, asymmetric coordination at higher movement frequencies provides a useful context in which to test whether transient challenge to right PMd disproportionately affects performance when coordination stability is placed under greater strain." As discussed in a previous comment, we also added the following justification for testing different movement frequencies in the 4th paragraph of the Introduction: "Manipulating movement frequency provides an additional task-demand manipulation. Higher movement frequencies increase the control demands of rhythmic tapping and amplify the behavioral costs associated with symbolic cueing and asymmetric coordination, thereby providing a more stringent test of whether PMd disruption impairs performance under high- demand conditions." The interpretation of the null effects would benefit from more explicit constraints on statistical power and detectable effect sizes. While Bayesian analyses are included, the manuscript does not clearly specify what magnitude of effect the study was adequately powered to detect. The design appears primarily powered for large effects, whereas smaller or moderate effects of rTMS are both plausible and commonly observed, particularly given known variability in behavioral responses to stimulation. Without defining these limits, the conclusions risk overstating what can be inferred from the null findings. Clarifying the range of detectable effect sizes and stating that the results primarily rule out large effects, rather than more subtle influences, would provide a more appropriately bounded interpretation.

We appreciate this point and agree that the null effects should be interpreted in relation to the magnitude of effects the study was designed to detect. We have clarified in the revised manuscript that the a priori power analysis was based on detecting relatively large effects, using estimates from prior work with a similar behavioral paradigm. Accordingly, the present design was primarily powered to detect large, readily observable stimulation-related effects and was less well suited to rule out smaller or more subtle behavioral influences of rTMS. We now state this more explicitly in both the Participants section and the Discussion so that the null findings are interpreted as constraining strong effects of transient right PMd challenge under the present protocol, rather than excluding more modest effects.

In response to this comment, we added the following sentence to the Participants section within Materials and Methods: "Thus, the present study was designed primarily to detect relatively large effects, and was less well suited to rule out smaller or more moderate stimulation-related influences." We also explicitly indicated we were powered only to detect relatively large effects at the end of our new section within the paragraph discussing the Bayesian analyses in the Discussion: "...the present findings constrain strong claims that transient disruption of right PMd is sufficient to impair asymmetric rhythmic movements or the management of increased cognitive load in rhythmic bimanual movement contexts, as described in our competing hypotheses (Figure 1), rather than excluding smaller or more subtle effects." Movement frequency clearly influences behavioral performance and appears to amplify asymmetry costs, yet it is not incorporated into the conceptual framework or predictions presented in the Introduction or Figure 1. As currently presented, frequency functions more as a statistical factor than a theoretically motivated variable. This raises questions about whether frequency is intended to reflect task difficulty, motor instability, or cognitive load, and whether it is expected to interact with response type in meaningful ways. Clarifying the intended role of frequency (e.g., control variable, theoretically relevant manipulation) would improve interpretability. If it is conceptually important, it should be incorporated into the hypotheses. If not, its role should be explicitly justified.

We agree that the role of movement frequency was not sufficiently articulated in the original manuscript. Movement frequency was included as an additional manipulation of task demand that was expected to increase the control demands of rhythmic tapping and amplify the costs associated with symbolic cueing and asymmetric coordination, particularly at higher frequencies (as we have shown in a previous related behavioural publication). This is consistent with prior work showing that asymmetric bimanual movements and symbolically cued movements place greater demands on domain-general cognitive resources than their spatially cued and symmetric counterparts.

In the revised manuscript, we now clarify this rationale in the Introduction and Figure 1 caption. Specifically, we added the following text to the last paragraph of the Introduction: "Manipulating movement frequency provides an additional task-demand manipulation. Higher movement frequencies increase the control demands of rhythmic tapping and amplify the behavioral costs associated with symbolic cueing and asymmetric coordination, thereby providing a more stringent test of whether PMd disruption impairs performance under high- demand conditions." Finally, we added the following text to the end of the caption for Figure 1: "For visual simplicity, the schematic illustrates the hypothesized contributions of response type and cue type to cognitive/control demands. Movement frequency is not depicted, but was included as an additional task-demand manipulation expected to increase control demands and amplify asymmetry-related costs at higher frequencies." Clarification of task and condition order is needed. In repeated measures motor tasks, practice, fatigue, or adaptation effects can meaningfully influence performance and may obscure subtle effects of stimulation, particularly in a design intended to detect potentially small neuromodulatory effects. It would be helpful to specify whether task components and conditions were fully counterbalanced across participants and, if not, to justify this choice or consider accounting for order effects analytically.

We appreciate this point and agree that the ordering of task components and conditions should be described more explicitly. We have revised the Methods to clarify that sham/rTMS session order was counterbalanced across participants, and that the order of cue type (spatial/symbolic) was also counterbalanced. However, response type was not counterbalanced within the rhythmic tapping task: participants always completed the asymmetric conditions before the symmetric conditions, and movement frequency always increased from 1.4 to 2.2 to 3 Hz within each condition. This choice was made for two reasons. First, fully counterbalancing all condition combinations would not have been feasible given the number of within-subject conditions. Second, we wanted to avoid participants starting at the highest movement frequency, as performance at 3 Hz was expected to be more difficult and participants may have benefited from first settling into the task at lower frequencies. We also prioritized presentation of the asymmetric conditions earlier in the post-stimulation period so that the theoretically most important comparison would occur well within the anticipated inhibitory window. We now acknowledge more explicitly that this fixed ordering may have introduced practice, fatigue, or adaptation effects, and therefore represents a limitation of the design. At the same time, because stimulation condition was tested in a sham-controlled within-subject design and post-stimulation testing was completed within the time window reported in Table 2, we do not think that condition order alone is likely to account for the null stimulation effect.

In response to this comment, we have made the following changes to the manuscript. First, in the Procedure section within Materials and Methods we now state: "The order of cue type (symbolic/spatial) was counterbalanced between participants. However, response type was not counterbalanced: all participants completed the asymmetric conditions before the symmetric conditions. Within each condition, movement frequency always increased from 1.4 to 2.2 to 3 Hz. This ordering was chosen to avoid beginning participants at the highest movement frequency and to ensure that the asymmetric conditions, which were of greatest theoretical interest, were completed earlier in the post-stimulation period." In the caption of Figure 2 we now explicitly state: "Within the rhythmic tapping task, cue type order was counterbalanced between participants, whereas response type order was fixed such that asymmetric conditions were completed before symmetric conditions. Within each condition, movement frequency always increased from 1.4 to 2.2 to 3 Hz. Within the bimanual 4-choice reaction time task, stimulus type condition order was counterbalanced between subjects." Finally, within the Limitations section of the Discussion we now state: "A related limitation is that condition order within the rhythmic tapping task was only partially counterbalanced. Although cue type order was counterbalanced, response type was fixed such that asymmetric conditions were always completed before symmetric conditions, and movement frequency always increased from 1.4 to 2.2 to 3 Hz. This design choice reduced the risk of participants beginning at the most difficult frequency and ensured that the asymmetric conditions were tested earlier in the post-stimulation period, but it may also have introduced practice, fatigue, or adaptation effects that could have influenced behavioral performance. However, substantial fatigue effects are unlikely given that the rhythmic tapping task was completed within approximately 13 min." The interpretation of symbolic versus spatial cues as a manipulation of cognitive load may be somewhat narrow. While symbolic cues likely increase cognitive demands, they also differ from spatial cues in abstraction, mapping requirements, and perceptual processing. As a result, it is difficult to attribute observed differences solely to cognitive load. The manuscript would benefit from a more cautious interpretation of this manipulation and earlier acknowledgment of alternative explanations, rather than limiting this discussion to the limitations section.

We appreciate this point. Our intention was not to imply that the symbolic versus spatial cue manipulation constitutes a pure or exclusive manipulation of cognitive load in an abstract sense. Rather, in the context of the present task and in line with prior work using the same behavioural paradigm, we use this contrast as an operational manipulation of increased cognitive/control demands during bimanual tapping. We agree, however, that the original wording could have been read too narrowly. In the revised manuscript, we have therefore softened the language throughout by replacing several instances of "cognitive load" with "cognitive control demands" and by clarifying this interpretation more explicitly in the Introduction. We retain the broader discussion of alternative explanations in the Limitations section, but do not think the present manuscript requires a more extensive theoretical treatment of the term itself, as the cue manipulation is operationalized explicitly within the task design description and hypothesis figure.

Minor Comments 1. Clarify how the FDI "hotspot" was identified and how motor threshold was determined.

We thank the reviewer for this suggestion. The hotspot and resting motor-threshold procedures were already described in the Methods, but we have revised this section to make the wording more explicit. Specifically, we now clarify that the FDI hotspot was defined as the scalp position at which single-pulse TMS most consistently elicited the largest MEPs in the resting FDI, and that resting motor threshold was defined as the minimum stimulation intensity required to evoke MEPs of at least 0.05 mV in 5 out of 10 consecutive trials.

2. Provide more detail on inclusion/exclusion criteria and handling of incorrect or missing response blocks across conditions.

We appreciate this suggestion and agree that the original manuscript did not describe the handling of exclusions, incorrect blocks, and missing observations as clearly as it could have. In the revised manuscript, we now clarify that exclusions were applied at the participant, block, and observation level depending on the nature of the issue. Specifically, we distinguish between participant-level exclusions due to insufficient task preparation or pervasive task-performance errors, block-level exclusion when isolated incorrect tapping blocks prevented computation of outcome measures, and observation-level exclusion of extreme outliers within condition. We also now state more explicitly that participants with only limited block-level errors were retained in the analyses with partial datasets, and that the mixed-effects models were selected in part because they allow inclusion of such partial data. In addition, we report the number of extreme outliers removed from the rhythmic tapping and within-hand intertap interval variability datasets, note that these removals were not concentrated in any single task condition, and clarify that re- running the primary analyses without outlier removal produced a similar overall pattern of results. This information has been added to the Quality Control section of the revised manuscript (within Materials and Methods).

In response to this comment, we made some small changes throughout the Quality Control section of Materials and Methods. We also added the following bigger block of text: "To reduce the risk of skewness biasing statistical models, and in line with previous statistical approaches (Denyer &Boyd, 2025) observations deemed extreme outliers (i.e., 3 SD above or below the mean) were removed from the dataset for each dependent measure. This led to partial datasets for some conditions. For the rhythmic tapping task, removal of extreme outliers resulted in the exclusion of 29 of 595 total observations (~5% of the dataset) for the mean absolute error of relative phase analysis and 29 of 595 total observations (~5% of the dataset) for the SD of absolute error analysis. For the supplementary within-hand intertap interval variability analysis, removal of extreme outliers resulted in the exclusion of 19 of 400 total observations (~4.8% of the dataset). Outlier detection was performed within each combination of movement frequency, response condition, cue condition, and stimulation condition, and inspection of the removed observations did not indicate concentration in any single task condition. Re-running analyses without outlier removal produced a similar overall pattern of results. The simple and choice reaction time tasks showed limited skewness, and therefore no observations were removed from these datasets before inferential analysis." 3. Consider including a schematic for the simple reaction time task comparable to the bimanual mapping figure.

We agree with this point and think it can increase transparency to the reader. Therefore, we have made a new Supplementary Figure 1 similar to Figure 2 which showcases the precise stimulus response mappings for the simple reaction time task.

4. Some figures with multiple conditions are difficult to interpret. Simplifying these or moving detailed breakdowns to supplementary material would improve clarity.

We appreciate this suggestion. To improve figure clarity and keep the main figures focused on the central experimental questions, we moved the simple RT results figure to the supplementary material as Supplementary Figure 4. Because the simple RT task served primarily as a control analysis rather than a primary outcome, we felt that presenting these data in the supplement improved the readability of the main manuscript without altering the interpretation of the results.

5. Visualizing key results as a function of movement frequency may aid interpretation.

We appreciate this suggestion. Rather than expanding the main figures to show all movement- frequency levels, which substantially reduced clarity in earlier drafts, we added a supplementary figure (Supplementary Figure 3) showing the focused 3 Hz analysis for mean absolute error. Because asymmetry-related costs were greatest at 3 Hz, this provides the most informative frequency-specific visualization of the key result while preserving readability in the main manuscript.

6. Provide clearer model specifications (fixed and random effects).

We thank the reviewer for this suggestion. The fixed and random effects for each model were already described in the original manuscript, but we have revised the wording slightly to make the model specifications more uniform across analyses. We also now explicitly describe Participant ID explicitly as a random intercept in the mixed-effects models.

7. Consider reporting effect sizes alongside statistical tests.

We appreciate this suggestion. For the mixed-effects models, we report estimated regression coefficients (β) and 95% confidence intervals, which provide the magnitude, direction, and precision of the observed effects. Because there is no single universally preferred standardized effect-size metric for all mixed-effects model terms, we have retained this reporting approach for the LMER results. For post hoc pairwise comparisons, standardized effect sizes are now reported as cohen's d where appropriate. We believe this provides an interpretable summary of both statistical significance and effect magnitude.

8. Ensure consistent terminology when referring to stimulation effects (e.g., avoid implying confirmed "disruption" without direct evidence).

We agree with the reviewer that disruption is overused in the manuscript given that it was no confirmed with a neural measure. In the revised manuscript, we re-checked all terminology to ensure that references to stimulation effects do not imply confirmed target disruption. Specifically, we softened wording in the Abstract, Introduction, Results, and Discussion so that our own manipulation is described in terms of stimulation applied over right PMd, transient challenge to right PMd, or intended reduction of excitability, rather than confirmed inhibition/disruption. Where stronger causal wording is retained, it is limited to discussion of prior literature rather than the present study.

9. Consider visualizing key results as a function of movement frequency. See response to minor comment 5.

10. Correct typographical errors (e.g., "cogntive").

We have re-checked all spelling including incidences of "cogntive" and endeavoured to fix all errors.
